# Characteristics and outcomes of 627 044 COVID-19 patients living with and without obesity in the United States, Spain, and the United Kingdom

**DOI:** 10.1038/s41366-021-00893-4

**Published:** 2021-07-15

**Authors:** Martina Recalde, Elena Roel, Andrea Pistillo, Anthony G. Sena, Albert Prats-Uribe, Waheed-Ul-Rahman Ahmed, Heba Alghoul, Thamir M. Alshammari, Osaid Alser, Carlos Areia, Edward Burn, Paula Casajust, Dalia Dawoud, Scott L. DuVall, Thomas Falconer, Sergio Fernández-Bertolín, Asieh Golozar, Mengchun Gong, Lana Yin Hui Lai, Jennifer C. E. Lane, Kristine E. Lynch, Michael E. Matheny, Paras P. Mehta, Daniel R. Morales, Karthik Natarjan, Fredrik Nyberg, Jose D. Posada, Christian G. Reich, Peter R. Rijnbeek, Lisa M. Schilling, Karishma Shah, Nigam H. Shah, Vignesh Subbian, Lin Zhang, Hong Zhu, Patrick Ryan, Daniel Prieto-Alhambra, Kristin Kostka, Talita Duarte-Salles

**Affiliations:** 1 Fundació Institut Universitari per a la recerca a l’Atenció Primària de Salut Jordi Gol i Gurina (IDIAPJGol), Barcelona, Spain; 2 Universitat Autònoma de Barcelona, Bellaterra, Spain; 3 Janssen Research & Development, Titusville, NJ USA; 4 Department of Medical Informatics, Erasmus University Medical Center, Rotterdam, The Netherlands; 5 Centre for Statistics in Medicine, NDORMS, University of Oxford, Oxford, UK; 6 Nuffield Department of Orthopaedics, Rheumatology, and Musculoskeletal Sciences, University of Oxford, Botnar Research Centre, Oxford, UK; 7 College of Medicine and Health, University of Exeter, St Luke’s Campus, Exeter, UK; 8 Faculty of Medicine, Islamic University of Gaza, Gaza, Palestine; 9 College of Pharmacy, Riyadh Elm University, Riyadh, Saudi Arabia; 10 Massachusetts General Hospital, Harvard Medical School, Boston, MA USA; 11 Nuffield Department of Clinical Neurosciences, University of Oxford, Oxford, UK; 12 Real-World Evidence, Trial Form Support, Barcelona, Spain; 13 Cairo University, Faculty of Pharmacy, Cairo, Egypt; 14 VA Informatics and Computing Infrastructure, VA Salt Lake City Health Care System, Salt Lake City, UT USA; 15 Department of Internal Medicine, University of Utah School of Medicine, Salt Lake City, UT USA; 16 Department of Biomedical Informatics, Columbia University, New York, NY USA; 17 Department of Epidemiology, Johns Hopkins School of Public, Baltimore, MD USA; 18 Pharmacoepidemiology, Regeneron Pharmaceuticals, Tarrytown, NY USA; 19 DHC Technologies co, Ltd, Beijing, China; 20 Division of Cancer Sciences, School of Medical Sciences, University of Manchester, Manchester, UK; 21 Tennessee Valley Healthcare System, Veterans Affairs Medical Center, Nashville, TN USA; 22 Department of Biomedical Informatics, Vanderbilt University Medical Center, Nashville, TN USA; 23 College of Medicine, The University of Arizona, Tucson, AZ USA; 24 Division of Population Health and Genomics, University of Dundee, Dundee, UK; 25 New York-Presbyterian Hospital, New York, NY USA; 26 School of Public Health and Community Medicine, Institute of Medicine, Sahlgrenska Academy, University of Gothenburg, Gothenburg, Sweden; 27 Department of Medicine, Stanford University, Palo Alto, CA USA; 28 Real World Solutions, IQVIA, Cambridge, MA USA; 29 Data Science to Patient Value Program, Department of Medicine, University of Colorado Anschutz Medical Campus, Aurora, CO USA; 30 College of Engineering, The University of Arizona, Tucson, AZ USA; 31 School of Population Medicine and Public Health, Chinese Academy of Medical Sciences & Peking Union Medical College, Beijing, China; 32 Melbourne School of Population and Global Health, The University of Melbourne, Melbourne, VIC Australia; 33 Institute of Health Management, Southern Medical University, Guangzhou, China; 34 Nanfang Hospital, Southern Medical University, Guangzhou, China; 35 The OHDSI Center at the Roux Institute, Northeastern University, Portland, ME USA

**Keywords:** Epidemiology, Public health

## Abstract

**Background:**

A detailed characterization of patients with COVID-19 living with obesity has not yet been undertaken. We aimed to describe and compare the demographics, medical conditions, and outcomes of COVID-19 patients living with obesity (PLWO) to those of patients living without obesity.

**Methods:**

We conducted a cohort study based on outpatient/inpatient care and claims data from January to June 2020 from Spain, the UK, and the US. We used six databases standardized to the OMOP common data model. We defined two non-mutually exclusive cohorts of patients *diagnosed* and/or *hospitalized* with COVID-19; patients were followed from index date to 30 days or death. We report the frequency of demographics, prior medical conditions, and 30-days outcomes (hospitalization, events, and death) by obesity status.

**Results:**

We included 627 044 (Spain: 122 058, UK: 2336, and US: 502 650) *diagnosed* and 160 013 (Spain: 18 197, US: 141 816) *hospitalized* patients with COVID-19. The prevalence of obesity was higher among patients *hospitalized* (39.9%, 95%CI: 39.8−40.0) than among those *diagnosed* with COVID-19 (33.1%; 95%CI: 33.0−33.2). In both cohorts, PLWO were more often female. Hospitalized PLWO were younger than patients without obesity. Overall, COVID-19 PLWO were more likely to have prior medical conditions, present with cardiovascular and respiratory events during hospitalization, or require intensive services compared to COVID-19 patients without obesity.

**Conclusion:**

We show that PLWO differ from patients without obesity in a wide range of medical conditions and present with more severe forms of COVID-19, with higher hospitalization rates and intensive services requirements. These findings can help guiding preventive strategies of COVID-19 infection and complications and generating hypotheses for causal inference studies.

## Introduction

Obesity is associated with increased mortality and is a well-known risk factor for chronic conditions, such as diabetes, hypertension, cardiovascular disease, and cancer [1, 2]. Due to its proinflammatory state that impairs the immune response, obesity has also been related to an increased risk of viral infections [3]. The novel coronavirus disease 2019 (COVID-19), caused by the severe acute respiratory syndrome coronavirus 2 (SARS-CoV-2), emerged in December 2019 in Wuhan, China, and rapidly spread around the world [4]. This new virus causes a respiratory tract infection with clinical manifestations ranging from asymptomatic/mild symptoms to severe illness requiring intensive services. Partly due to its similarities with other viral infections such as seasonal influenza or H1N1, people with obesity were soon labeled as “at-risk” individuals [5]. Since obesity is a worldwide public health priority, granular information on patients with COVID-19 and obesity is needed to guide preventive strategies as well as to generate hypotheses for etiological studies [6].

A review and meta-analysis of 75 studies reported that obesity is a risk factor for testing positive for SARS-CoV-2, for severe COVID-19 and for COVID-19 related mortality [7]. While undoubtedly relevant to the field, these studies mainly focused on exploring multiple risk factors related to COVID-19 and thus did not offer a detailed characterization of patients with COVID-19 living with obesity. For instance, an exhaustive description of the medical conditions and COVID-19 related outcomes, such as thromboembolic events, among these patients is lacking. Other current limitations include the susceptibility to collider bias of studies reporting “risk factors” of COVID-19 infection and progression due to sampling mechanisms (e.g., subsamples of tested or hospitalized populations) [8]. A large characterization study focussing exclusively on patients with COVID-19 living with obesity using real-world data from different health settings and countries could address the limitations of the previous evidence.

In this study, we aimed to describe and compare the demographics, medical conditions, and outcomes of COVID-19 patients living with obesity (PLWO) to those of COVID-19 patients living without obesity, in inpatient or outpatient settings.

## Methods

### Study design, setting, and data sources

We conducted a multinational cohort study using routinely collected healthcare data from January to June 2020 from Spain, the United Kingdom (UK), and the United States (US). This study was part of the “Characterizing Health Associated Risks, and Your Baseline Disease In SARS-COV-2 (CHARYBDIS)” study (protocol available for download at https://www.ohdsi.org/wp-content/uploads/2020/07/Protocol_COVID-19-Charybdis-Characterisation_V5.docx) designed by the Observational Health Data Sciences and Informatics (OHDSI) community. All data were standardized to the Observational Medical Outcomes Partnership (OMOP) Common Data Model (CDM) [9]. The OHDSI network maintains the OMOP-CDM, along with a wide range of tools developed by its members to facilitate analyses of mapped data [10]. Data results for this study were extracted on July, 16th, 2020.

We included primary, outpatient and inpatient care data from electronic health records (EHRs) and health insurance claims data from six databases. Data from Spain included the Information System for Research in Primary Care (SIDIAP), which includes primary linked to inpatient care data covering approximately 80% of the population in Catalonia, Spain [11]. The UK data covered the Clinical Practice Research Datalink (CPRD), with patients from over 600 general practices in the UK [12]. Data from the US included: Columbia University Irving Medical Center (CUIMC), covering New York-Presbyterian Hospital and its affiliated physician practices; IQVIA Open Claims, which are pre-adjudicated claims collected from office-based physicians and specialists covering over 300 million lives (~80% of the US population); the Stanford Medicine Research Data Repository (STARR-OMOP), with data from Stanford Health Care [13], and the United States Department of Veterans Affairs (VA-OMOP), covering the national Department of Veterans Affairs health care system which serves more than 9 million enrolled Veterans (of whom 93% are male). A more detailed description of the included data sources is available in Supplementary Appendix 1.

### Study participants

We included two non-mutually exclusive cohorts of patients: (1) all patients *diagnosed* with COVID-19 (clinical diagnosis and/or positive test for SARS-CoV-2), and (2) all patients *hospitalized* with a COVID-19 diagnosis. We considered clinical diagnoses in the definition of COVID-19 cases due to testing restrictions during the first months of the pandemic (e.g., in Spain) [14]. The diagnostic codes used are described in Supplementary Appendix 2. Patients *hospitalized* with COVID-19 were identified as those having a hospitalization episode along with a clinical diagnosis or positive SARS-CoV-2 test within a time window from 21 days prior to admission up to the end of their hospitalization. We chose this time window to include patients with a diagnosis prior to hospitalization and to allow for a record delay in test results or diagnoses [15]. We included individuals with at least one year of observation time prior to the index date to capture observed baseline characteristics. In the *diagnosed* cohort, the index date was defined as the date of the COVID-19 clinical diagnosis or the earliest test day registered within seven days of a first positive test, whichever occurred first. In the *hospitalized* cohort, the index date was the day of hospitalization. Patients were followed from the index date to the earliest of death, end of the observation period, or 30 days [16].

Both the *diagnosed* and *hospitalized* COVID-19 cohorts were stratified by obesity status: PLWO vs patients living without obesity (from now on, referred to as patients without obesity). Obesity was defined as having an ever-recorded obesity diagnosis (Supplementary Appendix 3) and/or a body mass index (BMI) measurement between 30 and 60 kg/m^2^ and/or a bodyweight measurement between 120 and 200 kg prior or at index date. We included upper cut-off thresholds to discard implausible observations. Patients without obesity were those who did not fulfill the obesity definition.

### Baseline characteristics and outcomes of interest

Demographics (sex and age) were obtained at the index date. More than 15 000 medical conditions from up to one year prior to the index date were identified based on the Systematized Nomenclature of Medicine (SNOMED) hierarchy, with all descendant codes included [15]. Specific definitions for comorbidities of particular interest were created; the detailed definitions of these variables can be consulted in Supplementary Appendix 3. We reported here a list of key comorbidities based on their prevalence in the cohorts of the participating sites, as well as on their clinical relevance to obesity and the COVID-19 research field [17].

Our 30-day outcomes of interest for the *diagnosed* cohort were hospitalization and fatality. For the *hospitalized* cohort, the 30-days outcomes were a requirement of intensive services (IS) (identified by a recorded mechanical ventilation and/or a tracheostomy and/or extracorporeal membrane oxygenation procedure), respiratory, cardiovascular, thromboembolic, and other events and fatality.

### Data analysis

We described the number of patients included and the prevalence of obesity in each database as well as the demographics, comorbidities, and outcomes as proportions (calculated by the number of persons within a given category, divided by the total number of persons) with their respective 95% confidence intervals (CIs) for each database, by obesity status. To calculate these proportions in each database, we established a minimum count required (of five individuals), to minimize the risk of re-identification of patients. To compare medical conditions across groups, we calculated standardized mean differences (SMDs) [18], which we summarized in Manhattan-style plots. The SMD can be used to compare the prevalence of a dichotomous variable between two groups and is independent of sample size [19]. A |SMD| > 0.1 indicates a meaningful difference in the prevalence of a given condition; in the context of this study, a SMD > 0.1 indicates a higher prevalence in PLWO, whereas a SMD < −0.1 indicates a higher prevalence among patients without obesity. This study was descriptive by nature and, therefore, statistical modeling was out of scope. Differences across the groups compared should not be interpreted as causal effects.

To ensure data privacy at all times, we employed a federated analysis approach [16]. Following a pre-specified analysis plan, a common analytical code for the whole CHARYBDIS study was developed for the OHDSI Methods library, available at https://github.com/ohdsi-studies/Covid19CharacterizationCharybdis, and was run locally in each database. Individual-level data remained within host institutions and only aggregate results from each database were provided to the research team and publicly shared. All the results reported in this paper and additional data are available for consultation at a dynamic and interactive website, which changes over time as new databases are added and/or results are updated to CHARYBDIS (https://data.ohdsi.org/Covid19CharacterizationCharybdis/).

We used R version 3.6 for data visualization. All the data partners obtained Institutional Review Board (IRB) approval or exemption to conduct this descriptive study.

## Results

### Prevalence of obesity

We included 627 044 *diagnosed* and 160 013 *hospitalized* patients with COVID-19 (Table 1). The *diagnosed* cohort consisted of 122 058 patients from Spain (SIDIAP), 2336 from the UK (CPRD), and 502 650 from the US (CUIMC: 8519; IQVIA-OpenClaims: 466 191; STARR-OMOP: 3328; VA-OMOP: 24 612). The *hospitalized* cohort included 18 197 patients from Spain (SIDIAP) and 141 816 from the US (CUIMC: 2600; IQVIA-OpenClaims: 133 091; STARR-OMOP: 615; VA-OMOP: 5510). Among *diagnosed* and *hospitalized* patients, 207 859 (33.1%; 95%CI: 33.0−33.2) and 63 866 (39.9%, 95%CI: 39.8−40.0) had obesity, respectively. In all databases, the prevalence of obesity was lower among *diagnosed* patients than among those *hospitalized*, with differences ranging from 5 (IQVIA-OpenClaims) to 16% (SIDIAP).

**Table 1:** Demographic characteristics of patients diagnosed and hospitalized with COVID-19 in each database, stratified by obesity status.

	SIDIAP (Spain)		CPRD (UK)		CUIMC (US)		IQVIA-Open Claims (US)		STARR-OMOP (US)		VA-OMOP (US)	
	With obesity	Without obesity	With obesity	Without obesity	With obesity	Without obesity	With obesity	Without obesity	With obesity	Without obesity	With obesity	Without obesity
Diagnosed with COVID-19												
All, *n*	36 409	85 649	976	1360	3446	5073	154 325	311 866	1157	2171	11 546	13 066
All, %* (95%CI)	29.8 (29.5−30.1)	70.2 (69.9−70.5)	41.8 (39.8−43.8)	58.2 (56.2−60.2)	40.5 (39.5−41.5)	59.5 (58.5−60.5)	33.1 (33.0−33.2)	66.9 (66.8-67)	34.8 (33.2−36.4)	65.2 (63.6−66.8)	46.9 (46.3−47.5)	53.1 (52.5−53.7)
Laboratory confirmed, % (95%CI)	39.2 (38.9−39.5)	27.2 (27−27.4)	51.5 (49.5−53.5)	50.3 (48.3−52.3)	66.7 (65.7−67.7)	65.5 (64.5−66.5)	–	–	24.4 (22.9−25.9)	28.6 (27.1−30.1)	–	–
Female sex, % (95%CI)	62.5 (62.2−62.8)	55.9 (55.6−56.2)	56.2 (54.2−58.2)	57.2 (55.2−59.2)	60.9 (59.9−61.9)	56.4 (55.3−57.5)	61.0 (60.9−61.1)	51.7 (51.6-51.8)	52.9 (51.2−54.6)	52.8 (51.1−54.5)	13.2 (12.8−13.6)	19.0 (18.5−19.5)
*Age, % (95%CI)*												
<18 years	1.2 (1.1−1.3)	4.7 (4.6−4.8)			1.2 (1−1.4)	3.5 (3.1−3.9)	1.0 (0.97−1.03)	3.9 (3.8−4)				
18−64 years	58.9 (58.6−59.2)	75.6 (75.4−75.8)	55.4 (53.4−57.4)	52.3 (50.3−54.3)	65.2 (64.2−66.2)	65.8 (64.8−66.8)	66.7 (66.6−66.8)	58.0 (57.9−58.1)	66.2 (64.6−67.8)	69.6 (68−71.2)	53.7 (53.1−54.3)	58.4 (57.8−59)
>65 years	40.0 (39.7−40.3)	19.6 (19.4−19.8)	44.7 (42.7−46.7)	47.7 (45.7−49.7)	33.6 (32.6−34.6)	30.6 (29.6−31.6)	32.3 (32.2−32.4)	38.2 (38.1−38.3)	33.8 (32.2−35.4)	30.4 (28.8−32)	46.3 (45.7−46.9)	41.6 (41−42.2)
Hospitalized with COVID-19												
All, *n*	8403	9794	–	–	1408	1192	50863	82228	274	341	2918	2592
All, % (95%CI)	46.2 (45.5−46.9)	53.8 (53.1−54.5)	–	–	54.2 (52.3−56.1)	45.8 (43.9−47.7)	38.2 (37.9−38.5)	61.8 (61.5−62.1)	44.6 (40.7−48.5)	55.4 (51.5−59.3)	53.0 (51.7−54.3)	47.0 (45.7−48.3)
Laboratory confirmed, % (95%CI)	76.0 (75.4−76.6)	72.9 (72.3−73.5)	–	–	88.5 (87.3−89.7)	91.6 (90.5−92.7)			10.6 (8.2−13)	18.3 (15.2−21.4)		
Female sex, % (95%CI)	51.2 (50.5−51.9)	39.9 (39.2−40.6)	–	–	54.7 (52.8−56.6)	39.8 (37.9−41.7)	55.0 (54.7−55.3)	43.5 (43.2−43.8)	51.5 (47.6−55.4)	49.0 (45.0−53.0)	6.6 (5.9−7.3)	4.4 (3.9−4.9)
*Age, % (95%CI)*												
<18 years	–	–	–	–	–	–	0.5 (0.5−0.5)	2.0 (1.9−2.1)	–	–	–	–
18−64 years	37.9 (37.2−38.6)	50.6 (49.9−51.3)	–	–	53.3 (51.4−55.2)	34.5 (32.7−36.3)	51.7 (51.4−52)	38.5 (38.2−38.8)	64.2 (60.4−68)	57.2 (53.3−61.1)	37.1 (35.8−38.4)	27.4 (26.2-28.6)
>65 years	62.1 (61.4−62.8)	49.4 (48.7−50.1)	–	–	46.7 (44.8−48.6)	65.6 (63.8−67.4)	47.8 (47.5−48.1)	59.5 (59.2−59.8)	35.8 (32.0−39.6)	42.8 (38.9−46.7)	62.9 (61.6−64.2)	72.6 (71.4−73.8)

*Notes.* * Proportion of patients with and without obesity among all patients (row percentage); - data not available or below the minimum cell count required (five individuals)

Abbreviations: *CI* confidence interval, *COVID-19* coronavirus disease 2019, *CPRD* Clinical Practice Research Datalink, *CUIMC* Columbia University Irving Medical Center, *SIDIAP* Information System for Research in Primary Care, *STARR-OMOP* Stanford Medicine Research Data Repository, *UK* United Kingdom, *US* United States, *VA-OMOP* United States Department of Veterans Affairs.

### Baseline demographics

The sex distribution (proportions and 95% CIs) of the patients are reported in Table 1. Aside from VA-OMOP, in the *diagnosed* cohort, patients with and without obesity were mostly female. The proportion of females was higher among PLWO compared to patients without obesity in SIDIAP (63% vs 56%), CUIMC (61% vs 56%), and IQVIA-OpenClaims (61% vs 52%), while in VA-OMOP the opposite was observed (13% vs 19%). No differences were observed in CPRD and STARR-OMOP. In the *hospitalized c*ohort, patients without obesity were predominantly male (female ranged from 40 to 49%, VA-OMOP: 4%) but PLWO still were more commonly female in all databases aside from VA-OMOP (range: 51−55%, VA-OMOP: 7%). Differences in the proportion of females between PLWO and patients without obesity ranged from 3 (VA-OMOP) to 15% (CUIMC).

**Fig. 1:**
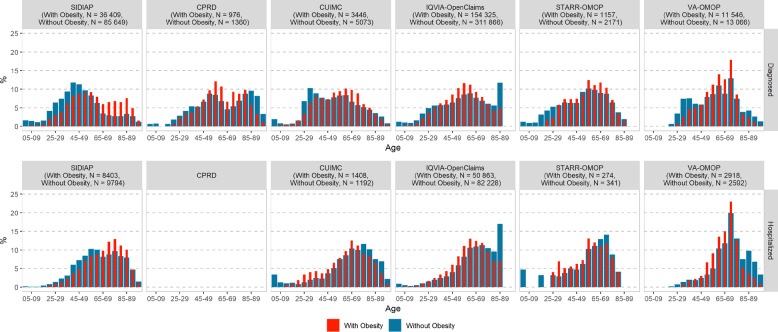
Distribution of age among patients living with and without obesity in each database, stratified by COVID-19 cohort type (diagnosed and hospitalized). CPRD Clinical Practice Research Datalink, COVID-19 coronavirus disease 2019, CUIMC Columbia University Irving Medical Center, SIDIAP Information System for Research in Primary Care, STARR-OMOP Stanford Medicine Research Data Repository, VA-OMOP United States Department of Veterans Affairs.

The age distribution in each database is summarized in Table 1 with proportions and their respective 95% CIs and in Fig. 1 with histograms. In the *diagnosed* cohort, PLWO were slightly older than those without obesity (i.e., the age distribution for PLWO was slightly skewed to the left compared to patients without obesity). This was particularly marked in SIDIAP, where 40% of the PLWO were aged above 65 years and only 20% were so without obesity. *Hospitalized* patients were older than those *diagnosed*. In the *hospitalized* cohorts, PLWO were fairly consistently younger than those without obesity (except for SIDIAP). The proportion of patients aged above 65 ranged from 36 to 63% for PLWO and from 43 to 73% for those without obesity.

### Baseline medical conditions

**Fig. 2:**
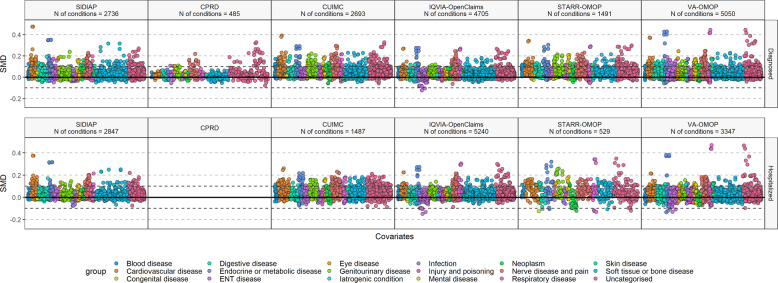
Standardized mean differences in conditions among patients living with obesity compared to patients living without obesity in each database, stratified by COVID-19 cohort type (diagnosed and hospitalized). SMD < 0 means the prevalence was greater in COVID-19 patients living without obesity, SMD > 0 means the prevalence was greater in COVID-19 patients living with obesity. COVID-19 coronavirus disease 2019, CPRD Clinical Practice Research Datalink, CUIMC Columbia University Irving Medical Center, SIDIAP Information System for Research in Primary Care, SMD standardized mean difference, STARR-OMOP Stanford Medicine Research Data Repository, VA-OMOP United States Department of Veterans Affairs.

We compared baseline medical conditions of PLWO to those of patients without obesity in the *diagnosed* and *hospitalized* cohorts using SMDs, which are summarized in Fig. 2. We depicted the SMDs of 485 (CPRD) to 5050 (VA-OMOP) medical conditions in the *diagnosed* cohort, and 529 (STARR-OMOP) to 5240 (IQVIA-OpenClaims) in the *hospitalized* cohort. In both cohorts, medical conditions were largely more frequent among PLWO than patients without obesity.

**Fig. 3:**
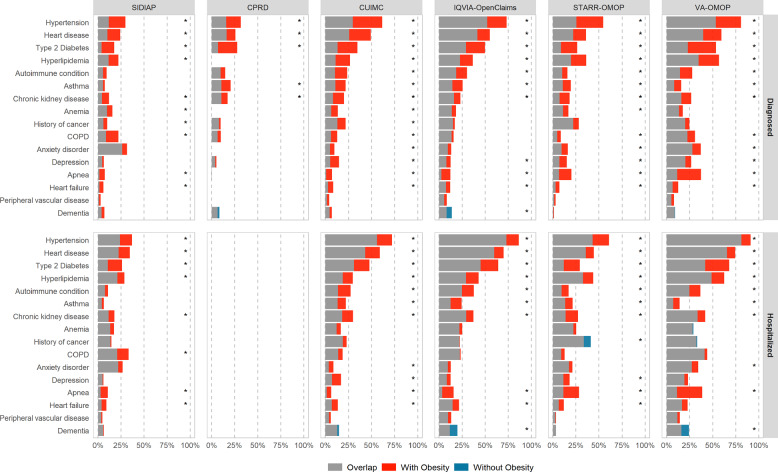
Comorbidities at baseline among patients living with obesity compared to patients living without obesity in each database, stratified by COVID-19 cohort type (diagnosed and hospitalized). Prevalence of comorbidities for COVID-19 patients living with obesity (red) and without obesity (blue) are depicted in overlapped horizontal bars. The gray color is the overlap between groups. E.g., in CPRD, 32% of COVID-19 patients living with obesity and 16% living without obesity have hypertension. Comorbidities with a meaningful difference (|SMD| > 0.1) between patients living with and without obesity are marked with an asterisk (*). COPD chronic obstructive pulmonary disease, COVID-19 coronavirus disease 2019, CPRD Clinical Practice Research Datalink, CUIMC Columbia University Irving Medical Center, SIDIAP Information System for Research in Primary Care, SMD standardized mean difference, STARR-OMOP Stanford Medicine Research Data Repository, VA-OMOP United States Department of Veterans Affairs.

The distribution of the selected key comorbidities is shown in Fig. 3, and the proportions with their respective 95% CIs and SMDs between PLWO and patients without obesity are available in Supplementary Appendices 4 and 5. In the *diagnosed* cohorts, PLWO consistently had a higher prevalence of comorbidities compared to those without obesity; these differences were meaningful (i.e., with a SMD > 0.1, which indicates a meaningfully higher prevalence among PLWO) for the majority of comorbidities across databases. For example, while the prevalence of hypertension for PLWO ranged from 30 to 32% in Europe (SIDIAP and CPRD) and from 55 to 81% in the US, in those without obesity it ranged from 12 to 16% and from 26 to 53%, respectively. The SMD for hypertension was above 0.1 in all databases. As in the *diagnosed* cohort, PLWO *hospitalized* with COVID-19 had a higher prevalence of comorbidities than those without obesity, and these differences were meaningful for the majority of comorbidities. However, the differences between groups were less obvious. For example, heart disease differed by 20% among those *diagnosed* in VA-OMOP (PLWO: 60%, without obesity: 40%) and by 9% among those *hospitalized* (PLWO: 74%, without obesity: 65%); although the SMD was still above 0.1 in all databases.

### 30-day outcomes of interest

**Fig. 4:**
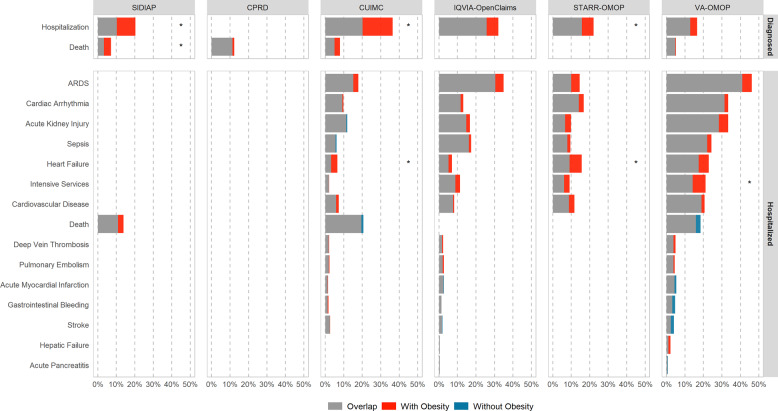
A comparison of 30-day events among patients living with and without obesity in each database, by COVID-19 cohort type (diagnosed and hospitalized). Proportion of outcomes for COVID-19 patients living with obesity (red) and without obesity (blue) are depicted in overlapped horizontal bars. The gray color is the overlap between groups. E.g., in the diagnosed cohort, 20 and 10% of patients living with and without obesity in SIDIAP, respectively, were hospitalized. Outcomes with a meaningful difference (|SMD| > 0.1) between patients living with obesity and patients without obesity are marked with an asterisk (*). ARDS acute respiratory distress syndrome, COVID-19 coronavirus disease 2019, CPRD Clinical Practice Research Datalink, CUIMC Columbia University Irving Medical Center, SIDIAP Information System for Research in Primary Care, SMD standardized mean difference, STARR-OMOP Stanford Medicine Research Data Repository, VA-OMOP United States Department of Veterans Affairs.

The distribution of 30-days outcomes is shown in Fig. 4, the proportions with their respective 95% CI and SMDs between PLWO and patients without obesity are available in Table 2. In the *diagnosed* cohorts, hospitalization rates were higher among PLWO than among those without obesity in all databases. For example, in SIDIAP the proportion of patients hospitalized was 20% for PLWO and 10% for patients without obesity. However, these differences were meaningful (SMD > 0.1) only in three databases: SIDIAP, CUIMC, and STARR-OMOP. In PLWO, fatality ranged from 5 to 12% and was higher than in patients without obesity in SIDIAP and CUIMC (7% vs 3% and 8% vs 5%, respectively), while in CPRD and VA-OMOP it was similar in both groups. SIDIAP was the only database with a meaningful difference in the proportion of fatality.

**Table 2:** Occurrence of 30-day events, in % (95%CI), among patients living with and without obesity in each database, by COVID-19 cohort type (diagnosed and hospitalized).

	SIDIAP (Spain)			CPRD (UK)			CUIMC (US)			IQVIA-Open Claims (US)			STARR-OMOP (US)			VA-OMOP (US)		
	With obesity	Without obesity	SMD	With obesity	Without obesity	SMD	With obesity	Without obesity	SMD	With obesity	Without obesity	SMD	With obesity	Without obesity	SMD	With obesity	Without obesity	SMD
*Patients diagnosed*, *n*	36 409	854 649	NA	976	1360	NA	3446	5073	NA	154 325	311 866	NA	1157	2171	NA	11 546	13 066	NA
Hospitalization	20.3 (19.9−20.7)	10.3 (10.2−10.4)	0.20	–	–	–	36.4 (34.8−38.0)	20.2 (19.1−21.3)	0.26	32.1 (31.9−32.3)	25.8 (25.6−26.0)	0.10	22.1 (19.7−24.5)	15.8 (14.3−17.3)	0.11	16.6 (15.9−17.3)	12.8 (12.2−13.4)	0.08
Death	7.1 (6.8−7.4)	3.3 (3.3−3.3)	0.12	12.3 (10.2−14.4)	11.3 (9.6−13.0)	0.02	8.0 (7.1−8.9)	5.0 (4.4−5.6)	0.09	–	–	–	–	–	–	5.1 (4.7−5.5)	4.8 (4.4−5.2)	0.01
*Patients hospitalized*, *n*	8403	9794	NA	–	–	NA	1408	1192	NA	50 863	8228	NA	274	341	NA	2918	2592	NA
Intensive service requirement	–	–	–	–	–	–	2.3 (1.5−3.1)	2.0 (1.2−2.8)	0.01	13.0 (12.7−13.3)	9.7 (9.1−10.3)	0.06	9.1 (5.7−12.5)	6.2 (3.6−8.8)	0.08	21.9 (20.4−23.4)	14.5 (13.1−15.9)	0.13
Death	13.9 (13.2−14.6)	10.9 (10.3−11.5)	0.06	–	–	–	19.5 (17.4−21.6)	20.6 (18.3−22.9)	−0.02	–	–	–	–	–	–	15.9 (14.6−17.2)	18.4 (16.9−19.9)	−0.05
*Cardiovascular events*																		
Acute myocardial infarction	–	–	–	–	–	–	1.3 (0.7−1.9)	1.1 (0.5−1.7)	0.01	2.1 (2.0−2.2)	2.2 (1.9−2.5)	−0.01	–	–	–	5.5 (4.7−6.3)	6.9 (5.9−7.9)	−0.03
Cardiac arrhythmia*	–	–	–	–	–	–	9.8 (8.2−11.4)	9.3 (7.7−10.9)	0.01	13.1 (12.8−13.4)	11.7 (11.0−12.4)	0.03	16.8 (12.4−21.2)	14.1 (10.4−17.8)	0.05	33.4 (31.7−35.1)	31.4 (29.6−33.2)	0.03
Heart failure*	–	–	–	–	–	–	6.5 (5.2−7.8)	3.1 (2.1−4.1)	0.11	7.0 (6.8−7.2)	5.1 (4.6−5.6)	0.06	15.7 (11.4−20.0)	9.1 (6.0−	0.14	22.9 (21.4−24.4)	17.4 (15.9−18.9)	0.10
Stroke	–	–	–	–	–	–	2.6 (1.8−3.4)	2.5 (1.6−3.4)	0.00	1.7 (1.6−1.8)	1.9 (1.6−2.2)	−0.01	–	–	–	2.4 (1.8−3.0)	4.0 (3.2−4.8)	−0.06
Total cardiovascular disease events	–	–	–	–	–	–	7.3 (5.9−8.7)	5.9 (4.6−7.2)	0.04	8.2(8.0−8.4)	7.6 (7.0−8.2)	0.02	11.7 (7.9−15.5)	8.8 (5.8−11.8)	0.07	20.6 (19.1−22.1)	19.0 (17.5−20.5)	0.03
*Thromboembolic events*																		
Deep vein thrombosis	–	–	–	–	–	–	2.1 (1.4−2.8)	1.8 (1.0−2.6)	0.02	2.2 (2.1−2.3)	1.7 (1.4−2.0)	0.03	–	–	–	4.9 (4.1−5.7)	3.8 (3.1−4.5)	0.04
Pulmonary embolism	–	–	–	–	–	–	2.2 (1.4−3.0)	1.8 (1.0−2.6)	0.02	1.8 (1.7−1.9)	1.5 (1.2−1.8)	0.03	–	–	–	3.8 (3.1−4.5)	3.3 (2.6−4.0)	0.02
*Other events*																		
Acute kidney injury*	–	–	–	–	–	–	18.1 (16.1−20.1)	25.4 (22.9−27.9)	−0.01	11.1 (10.8−11.4)	10.4 (9.7−11.1)	0.04	8.4 (5.1−11.7)	9.1 (6.0−12.2)	0.08	17.1 (15.7−18.5)	18.6 (17.1−20.1)	0.08
Acute pancreatitis	–	–	–	–	–	–	–	–	–	0.2 (0.2−0.2)	0.2 (0.1−0.3)	0.00	–	–	–	0.4 (0.2−0.6)	0.7 (0.4−1.0)	−0.02
ARDS*	–	–		–	–		17.9 (15.9−19.9)	15.2 (13.2−17.2)	0.05	34.9 (34.5−35.3)	30.5 (29.5−31.5)	0.07	14.6 (10.4−18.8)	10.0 (6.8−13.2)	0.10	46.2 (44.4−48.0)	41.0 (39.1−42.9)	0.07
Gastrointestinal bleeding	–	–	–	–	–	–	1.7 (1.0−2.4)	1.0 (0.4−1.6)	0.02	1.3 (1.2-−1.4)	1.3 (1.1−1.5)	0.00	–	–	–	3.1 (2.5−3.7)	4.7 (3.9−5.5)	−0.06
Hepatic failure	–	–	–	–	–	–	0.8 (0.3−1.3)	–	–	0.2 (0.2−0.2)	0.2 (0.1−0.3)	0.00	–	–	–	1.6 (1.1−2.1)	0.8 (0.5−1.1)	0.06
Sepsis*	–	–	–	–	–	–	5.6 (4.4−6.8)	6.0 (4.7−7.3)	−0.02	17.4 (17.1−17.7)	16.2 (15.4−17.0)	0.02	9.5 (6.0−13.0)	7.9 (5.0−10.8)	0.04	24.3 (22.7−25.9)	22.0 (20.4−23.6)	0.04

*Notes*. - data not available or below the minimum cell count required (five individuals); events marked with an * were recorded only during hospitalization. SMD < 0 means the prevalence was greater in COVID-19 patients living without obesity, SMD > 0 means the prevalence was greater in COVID-19 patients living with obesity. A SMD > |0.1| indicates a meaningful difference in the prevalence of a given condition

Abbreviations: *ARDS* acute respiratory distress syndrome, *CI* confidence interval, *COVID-19* coronavirus disease 2019, *CPRD* Clinical Practice Research Datalink, *CUIMC* Columbia University Irving Medical Center, *NA* not applicable, *SIDIAP* Information System for Research in Primary Care, *SMD* standardized mean difference, *STARR-OMOP* Stanford Medicine Research Data Repository, *UK* United Kingdom, *US* United States, *VA-OMOP* United States Department of Veterans Affairs.

Overall, in the *hospitalized* cohort, PLWO more frequently had adverse events occurring in the 30 days after the index date than patients without obesity. For example, PLWO required IS and presented with ARDS more frequently than patients without obesity in the largest databases: IQVIA-OpenClaims (IS: 13% vs 10%; ARDS: 35% vs 31%) and VA-OMOP (IS: 22% vs 15%; 46% vs 41%), whereas in CUIMC and STARR-OMOP percentages were similar. VA-OMOP was the only database with a meaningful difference in the proportion of IS. Similarly, heart failure was also more frequent among PLWO than among patients without obesity in CUIMC: 7% vs 3%, IQVIA-OpenClaims: 7% vs 5%, STARR-OMOP: 16% vs 9%, and VA-OMOP: 23% vs 17%), these differences were meaningful in CUIMC and STARR-OMOP. Sepsis, cardiac arrhythmia, and cardiovascular disease events were slightly more frequent among PLWO, although SMDs were below 0.1 in all databases. Acute kidney injury was the only outcome that was more frequent among patients without obesity; however, this difference was not meaningful in any database. As for fatality, there were no consistent nor meaningful differences between PLWO and patients without obesity in the *hospitalized cohort:* while it was higher for PLWO in SIDIAP (14% vs 11%), there were no differences in CUIMC (20% vs 21%) nor in VA-OMOP (16% vs 18%).

## Discussion

In this large cohort study including 627 044 COVID-19 patients from Spain, the UK, and the US, we found that the prevalence of obesity was higher among COVID-19 patients *hospitalized* (40%) compared to those *diagnosed* (31%). PLWO *diagnosed* and *hospitalized* with COVID-19 were more commonly female, and those hospitalized were younger than patients without obesity. The extraction of more than 15 000 medical conditions revealed PLWO were not only more prone to have obesity-related comorbidities, such as hypertension, heart disease, and type 2 diabetes but also to more than a thousand different health conditions. After 30-days of follow-up, PLWO presented with higher hospitalization rates and intensive services requirements, although these differences were only meaningful in some databases.

Our study has several strengths, such as its large amount of data. By bringing together harmonized data using a federated approach, we have conducted a large-scale study while respecting the confidentiality of patient records. The international approach of this study is a strong asset given that we are investigating the intersection of two major global threats, namely the obesity epidemic and the COVID-19 pandemic. The former, together with the diverse healthcare settings and populations described in this study, increase the generalizability of our findings. Further, we provide a wide overview of the characteristics and outcomes of patients with and without obesity, using data visualization tools to summarize large amounts of medical data. This exhaustive characterization goes far beyond prior studies reporting few comorbidities and supports the generation of new hypotheses that can be tested in future studies. In addition, for the sake of transparency and reproducibility, we have made methods, tools, and all results publicly available. As CHARYBDIS is an ongoing study, results (included longer follow-up time) will be updated and new studies focussing on obesity could be conducted. All of the above has been accomplished through the coordinated efforts of the OHDSI community to provide a rapid response to the COVID-19 pandemic.

Our study also has limitations. First, we cannot exclude a selection bias of COVID-19 cases due to underreporting in the context of testing restrictions and asymptomatic or paucisymptomatic cases that usually do not seek medical care. Additionally, testing policies have varied across countries and time depending on the course of the pandemic. Nevertheless, the inclusion of patients clinically diagnosed (not tested) in different settings likely provided consistency to our data, although it might have incurred in false positives. Second, we did not have information on BMI as a continuous variable, which prevented us from investigating the impact of different categories of obesity in COVID-19 outcomes. This might explain the higher proportion of comorbidities and outcomes observed in the US databases, as PLWO from the US might have higher BMIs than those from Europe [20]. In addition, our definition of obesity included diagnoses and measurements recorded at any time prior to or at the index date, and therefore some individuals might have been misclassified due to changes in BMI since the most recently recorded status. However, previous evidence shows that BMI trajectories in adults are relatively stable, with a tendency to increase with age [21]. Therefore adults with obesity are likely to still have obesity over time. Finally, this study was underpinned by routinely collected data which can raise concerns about the quality of the data. Some databases are prone to oversampling certain groups of people as a result of how these data are captured (e.g., the Veterans Affairs system historically serves more men than women, routine claims data may only reflect health outcomes in commercially insured populations, etc.). Obesity, comorbidities, and outcomes were assessed based on having a record of a condition/measurement, therefore they may be underestimated. In addition, outcomes such as hospitalization or intensive services requirements are also influenced by factors external to the patient’s condition (i.e., bed availability, criteria for admission), which might differ across databases. Even still, the consistency of our findings across databases that differ by setting and country lends credence to the generalizability of our findings.

Given the prevalence of obesity in Spain (24%), the UK (27%), and the US (37%), a high proportion of PLWO among COVID-19 cases was expected [20]. However, the prevalence of obesity among diagnosed COVID-19 patients was higher than the general population in four databases: SIDIAP (Spain): 30%; CPRD (UK): 42%; CUIMC and VA-OMOP (US): 41 and 47%, respectively, which is suggestive of an increased risk of diagnosis in PLWO. In addition, the prevalence of obesity was higher in hospitalized COVID-19 patients, with an overall prevalence of obesity of 40%, which is in line with three cohort studies from the US that reported that 40, 42, and 48% of inpatients were living with obesity [17, 22, 23]. A large meta-analysis of observational studies reported that obesity is associated with a higher risk of testing positive for SARS-CoV-2 or being diagnosed with COVID-19 as well as of being hospitalized with COVID-19 [7]. While this could be due to an increased vulnerability to SARS-CoV-2 in PLWO, other hypotheses should be considered in future studies. On the one hand, individuals with obesity could be more likely to seek care and be tested for SARS-CoV-2 since they are (presumably) a high-risk population, have multiple comorbidities, and are more prone to respiratory symptoms due to their compromised pulmonary function [2, 7]. On the other hand, given the fact that obesity disproportionately affects disadvantaged populations, potential differential exposures across subpopulation groups should also be explored (e.g., differential occupational risks) [2].

Women predominated among hospitalized patients with obesity, even though obesity rates are similar in both sexes in the three countries [20]. Although male sex is a well-established risk factor for COVID-19-related hospitalization and death, little is known about the role of obesity on COVID-19 outcomes stratified by sex [14, 23–25]. Recent studies addressing this issue in secondary analyses have reported inconsistent results. A study conducted among UK Biobank participants found that the impact of BMI in COVID-19-related death was higher among females compared to men, while others have found a higher effect among males, opposite effects of sex in different age strata or null differences [26–29]. Thus, the intersection between sex/gender and obesity in relation to COVID-19-outcomes warrants further investigation. Because sex-stratification was beyond the pre-specified analysis plan of our study, we were unable to report our results by sex, which could have provided valuable insights on the matter. We intend, however, to address this issue in upcoming studies from the CHARYBDIS project.

We also found that *hospitalized* PLWO were younger than those without obesity. Although younger individuals have less risk of infections and complications than older people due to having fewer comorbidities and a stronger immune system, this is not the case for those with obesity [2, 7, 30–32]. Some authors have postulated that PLWO younger than 60 years could have a greater risk of severe COVID-19 outcomes [33]. PLWO also had many more comorbidities than patients without obesity. Unsurprisingly, the highest differences were observed in obesity-related conditions, such as hypertension, diabetes, and heart disease, which have been identified as risk factors for severe COVID-19 outcomes [14, 17, 25, 34, 35]. However, as our findings revealed, PLWO with COVID-19 differ from patients without obesity in a wider range of medical conditions than previously described. Future etiological studies aiming to disentangle the effect of obesity in COVID-19 outcomes should have this information present and consider data-driven techniques to account for confounding, such as propensity score estimation and its adjustment methods [18].

Finally, PLWO experienced adverse events more frequently than those without obesity, particularly hospitalization and the requirement of intensive services. Certainly, our results must be interpreted carefully considering the differences in demographics and comorbidities between these groups. Interestingly, in patients hospitalized, we did not observe clear differences in fatality between patients with and without obesity. While two meta-analyses reported that obesity is associated with a higher risk of COVID-19 related mortality; other large observational studies from the US and the UK using finer categories of BMI only found an association with mortality for morbid obesity (BMIs ≥ 35 kg/m_2_ or ≥40 kg/m_2_) [7, 25, 28, 29, 34]. Given the scarcity of evidence regarding the frequency of specific adverse events during hospitalization among PLWO, our findings are of special interest to the field and should be addressed in upcoming etiological studies.

In this large international cohort, we showed that among COVID-19 cases, PLWO were more likely to be female, have more comorbidities, and worse outcomes than patients without obesity. The prevalence of obesity was higher among hospitalized patients with COVID-19 compared to patients diagnosed with COVID-19. Our results may be useful in guiding clinical practice and aid future preventative strategies for patients living with obesity, as well as providing useful data to support subsequent etiological studies focussed on obesity and COVID-19.

## Supplementary information

Supplementary Material

## Data sharing statement

Analyses were performed locally in compliance with all applicable data privacy laws. Although the underlying data is not readily available to be shared, authors contributing to this paper have direct access to the data sources used in this study. All results (e.g., aggregate statistics, not presented at a patient-level with redactions for minimum cell count) are available for public inquiry. These results are inclusive of site-identifiers by contributing data sources to enable interrogation of each contributing site. All analytic code and result sets are made available at: https://github.com/ohdsi-studies/Covid19CharacterizationCharybdis.
