# Supplementary material for: Characteristics and outcomes of 627 044 COVID-19 patients living with and without obesity in the United States, Spain, and the United Kingdom
Source: Int J Obes (Lond). 2021 Jul 15;45(11):2347–57. doi: 10.1038/s41366-021-00893-4 (PMC8281807; doi:10.1038/s41366-021-00893-4)
Supplement: Supplementary file 1 — Supplementary Material [file 41366_2021_893_MOESM1_ESM.docx]

**Supplementary Material: Characteristics and outcomes of 627 044 COVID-19 patients living with and without obesity in the United States, Spain, and the United Kingdom**

Martina Recalde MPH^1,2^*, Elena Roel MD MPH^1,2^*, Andrea Pistillo MSc^1^, Anthony G. Sena BA^3, 4^, Albert Prats-Uribe MD MPH^5^, Waheed-Ul-Rahman Ahmed^6,7^, Heba Alghoul MD^8^, Thamir M Alshammari PhD^9^, Osaid Alser MD^10^, Carlos Areia MSc^11^, Edward Burn PhD^1, 5^, Paula Casajust MSc^12^, Dalia Dawoud PhD^13^, Scott L. DuVall PhD^14, 15^, Thomas Falconer MS^16^, Sergio Fernández-Bertolín BSc^1^, Asieh Golozar MD PhD MHS MPH^17, 18^, Mengchun Gong MD^19^, Lana Yin Hui Lai PhD^20^, Jennifer C.E. Lane MRCS^6^ , Kristine E. Lynch PhD^14, 15^, Michael E. Matheny MD MPH^21, 22^, Paras P Mehta BA^23^, Daniel R. Morales MD PhD^24^, Karthik Natarjan PhD^16, 25^, Fredrik Nyberg MD PhD^26^, Jose D. Posada PhD^27^, Christian G. Reich MD PhD^28^, Peter Rijnbeek PhD^4^, Lisa M. Schilling MD MSPH^29^, Karishma Shah MBBS^6^, Nigam H. Shah MBBS PhD^27^, Vignesh Subbian PhD^30^, Lin Zhang MD PhD^31, 32^, Hong Zhu PhD^33, 34^, Patrick Ryan PhD^3, 16^, Daniel Prieto-Alhambra Prof^5^, Kristin Kostka MPH^28,35^**, Talita Duarte-Salles PhD^1^**

*Joint first authors

**Joint last authors

1. Fundació Institut Universitari per a la recerca a l’Atenció Primària de Salut Jordi Gol i Gurina (IDIAPJGol), Barcelona, Spain

2. Universitat Autònoma de Barcelona, Spain

3. Janssen Research & Development, Titusville, NJ, USA

4. Department of Medical Informatics, Erasmus MC, Rotterdam, The Netherlands

5. Centre for Statistics in Medicine, NDORMS, University of Oxford

6. Nuffield Department of Orthopaedics, Rheumatology, and Musculoskeletal Sciences, University of Oxford, Botnar Research Centre, Windmill Road, Oxford, OX3 7LD, UK.

7. College of Medicine and Health, University of Exeter, St Luke’s Campus, Heavitree Road, Exeter, EX1 2LU, UK.

8. Faculty of Medicine, Islamic University of Gaza, Palestine

9. College of Pharmacy, Riyadh Elm University, Riyadh, Saudi Arabia

10. Massachusetts General Hospital, Harvard Medical School, Boston, 02114, Massachusetts, USA

11. Nuffield Department of Clinical Neurosciences, University of Oxford, UK

12. Real-World Evidence, Trial Form Support, Barcelona, Spain

13. Cairo University, Faculty of Pharmacy, Cairo, Egypt

14. VA Informatics and Computing Infrastructure, VA Salt Lake City Health Care System, Salt Lake City, UT, USA

15. Department of Internal Medicine, University of Utah School of Medicine, Salt Lake City, UT, USA

16. Department of Biomedical Informatics, Columbia University, New York, NY, USA

17. Department of Epidemiology, Johns Hopkins School of Public, Baltimore MD, USA

18. Pharmacoepidemiology, Regeneron Pharmaceuticals, NY, US

19. DHC Technologies co, ltd., Beijing, China

20. Division of Cancer Sciences, School of Medical Sciences, University of Manchester

21. Tennessee Valley Healthcare System, Veterans Affairs Medical Center, Nashville, TN, USA

22. Department of Biomedical Informatics, Vanderbilt University Medical Center, Nashville, TN, USA

23. College of Medicine, The University of Arizona, Tucson, AZ, USA

24. Division of Population Health and Genomics, University of Dundee, UK

25. New York-Presbyterian Hospital, New York, NY, US

26. School of Public Health and Community Medicine, Institute of Medicine, Sahlgrenska Academy, University of Gothenburg, Gothenburg, Sweden

27. Department of Medicine, Stanford University, Palo Alto, California, USA

28. Real World Solutions, IQVIA, Cambridge, MA, USA

29. Data Science to Patient Value Program, Department of Medicine, University of Colorado Anschutz Medical Campus

30. College of Engineering, The University of Arizona, Tucson, AZ, USA

31. School of Population Medicine and Public Health, Chinese Academy of Medical Sciences & Peking Union Medical College

32. Melbourne School of Population and Global Health, The University of Melbourne

33. Institute of Health Management, Southern Medical University, Guangzhou, China

34. Nanfang Hospital, Southern Medical University, Guangzhou, China

35. The OHDSI Center at the Roux Institute, Northeastern University, Portland, ME, USA

**Supplementary material**

[Appendix 1. Description of databases](#_heading=h.oz51tqwr8f06)

Appendix 2. Definitions and codes used to identify COVID-19 cases

Appendix 3. Definitions and codes used for obesity and other comorbidities

Appendix 4. Table 3: Selected comorbidities, in % (95%CI) among patients living with and without obesity diagnosed with COVID-19, by database

Appendix 5. Table 4: Selected comorbidities, in % (95%CI) among patients living with and without obesity hospitalized with COVID-19, by database

####

#### Appendix 1. Description of databases

| **Institution Name** | **Database** | **Database Description** | **Country** |
| --- | --- | --- | --- |
| Janssen Research & Development | The Clinical Practice Research Datalink (CPRD) | The Clinical Practice Research Datalink (CPRD) is a governmental not-for-profit research service jointly funded by the NHS National Institute for Health Research (NIHR) and the Medicines and Healthcare products Regulatory Agency (MHRA) a part of the Department of Health United Kingdom (UK). CPRD consists of data collected from UK primary care for all ages. This includes conditions observations measurements and procedures that the general practitioner is made aware of in addition to any prescriptions as prescribed by the general practitioner. In addition to primary care there are also linked secondary care records for a small number of people. The major data elements contained within this database are outpatient prescriptions given by the general practitioner (coded with Multilex codes) and outpatient clinical referral immunization or test events that the general practitioner knows about (coded in Read or ICD10 or LOINC codes). The database also contains the patients’ year of births and any date of deaths. | United Kingdom |
| IDIAPJGol | The Information System for Research in Primary Care (SIDIAP) | The Information System for Research in Primary Care (SIDIAP; www.sidiap.org) is a primary care records database that covers approximately 80% of the population of Catalonia North-East Spain. Healthcare is universal and tax-payer funded in the region and primary care physicians are gatekeepers for all care and responsible for repeat prescriptions. | Spain |
| Stanford | Stanford Medicine Research Data Repository (STARR-OMOP) | A clinical data warehouse containing live Epic data from Stanford Health Care the Stanford Children’s Hospital the University Healthcare Alliance and Packard Children's Health Alliance clinics.  Reference: Datta S Posada J Olson G *et al.* A new paradigm for accelerating clinical data science at Stanford Medicine. *arXiv* 2020; published online March 17. http://arxiv.org/abs/2003.10534 (accessed Aug 20 2020). | United States |
| Columbia | Columbia University Irving Medical Center (CUIMC) | The clinical data warehouse of New York-Presbyterian Hospital/Columbia University Irving Medical Center New York NY based on its current and previous electronic health record systems with data spanning over 30 years and including over 6 million patients | United States |
| IQVIA | Open Claims | Pre-adjudicated claims covering over 300 Million lives (~80% of the US) collected from office-based physicians and specialists via office management software and clearinghouse switch sources for the purpose of reimbursement. | United States |
| Department of Veterans Affairs | V- OMOP | VA-OMOP data reflects the national Department of Veterans Affairs health care system which is the largest integrated provider of medical and mental health services in the United States. Care is provided at 170 VA Medical Centers and 1 063 outpatient sites serving more than 9 million enrolled Veterans each year. | United States |

#### Appendix 2. Definitions and codes used to identify COVID-19 cases

The below tables summarise the concepts used to identify patients diagnosed with COVID-19 . The full description of the logic used to identify patients diagnosed and hospitalized is provided at <https://atlas.ohdsi.org/#/cohortdefinition/200> and <https://atlas.ohdsi.org/#/cohortdefinition/197> respectively.

**COVID-19 condition codes**

| **Id** | **Name** | **Vocabulary** |
| --- | --- | --- |
| 756023 | Acute bronchitis due to COVID-19 | OMOP Extension |
| 756044 | Acute respiratory distress syndrome (ARDS) due to COVID-19 | OMOP Extension |
| 756061 | Asymptomatic COVID-19 | OMOP Extension |
| 756031 | Bronchitis due to COVID-19 | OMOP Extension |
| 439676 | Coronavirus infection | SNOMED |
| 37311061 | Disease caused by 2019-nCoV | SNOMED |
| 4100065 | Disease due to Coronaviridae | SNOMED |
| 37310284 | Encephalopathy caused by 2019 novel coronavirus | SNOMED |
| 37310283 | Gastroenteritis caused by 2019 novel coronavirus | SNOMED |
| 4248811 | Healthcare associated severe acute respiratory syndrome | SNOMED |
| 756081 | Infection of lower respiratory tract due to COVID-19 | OMOP Extension |
| 37310286 | Infection of upper respiratory tract caused by 2019 novel coronavirus | SNOMED |
| 45763594 | Middle East respiratory syndrome | SNOMED |
| 37310287 | Myocarditis caused by 2019 novel coronavirus | SNOMED |
| 37310254 | Otitis media caused by 2019 novel coronavirus | SNOMED |
| 37310285 | Pneumonia caused by 2019 novel coronavirus | SNOMED |
| 37016927 | Pneumonia caused by Human coronavirus | SNOMED |
| 40479642 | Pneumonia due to Severe acute respiratory syndrome coronavirus | SNOMED |
| 756039 | Respiratory infection due to COVID-19 | OMOP Extension |
| 320651 | Severe acute respiratory syndrome | SNOMED |
| 37396171 | Severe acute respiratory syndrome of upper respiratory tract | SNOMED |
| 37311060 | Suspected disease caused by 2019-nCoV | SNOMED |

**COVID-19 specific testing - Positive**

| **Id** | **Name** | **Vocabulary** |
| --- | --- | --- |
| 37310282 | 2019 novel coronavirus detected | SNOMED |

**COVID-19 specific testing (note these required a corresponding value as concept of: Detected Positive or Present)**

| **Id** | **Name** | **Vocabulary** |
| --- | --- | --- |
| 37310255 | Detection of 2019 novel coronavirus using polymerase chain reaction technique | SNOMED |
| 700360 | Infectious agent detection by nucleic acid (DNA or RNA); severe acute respiratory syndrome coronavirus 2 (SARS-CoV-2) (Coronavirus disease [COVID-19]) amplified probe technique | CPT4 |
| 37310258 | Measurement of 2019 novel coronavirus antibody | SNOMED |
| 37310257 | Measurement of 2019 novel coronavirus antigen | SNOMED |
| 756055 | Measurement of Severe acute respiratory syndrome coronavirus 2 (SARS-CoV-2) | OMOP Extension |
| 586310 | Measurement of Severe acute respiratory syndrome coronavirus 2 (SARS-CoV-2) Genetic material using Molecular method | OMOP Extension |
| 704991 | Measurement of Severe acute respiratory syndrome coronavirus 2 (SARS-CoV-2) in Blood | OMOP Extension |
| 756029 | Measurement of Severe acute respiratory syndrome coronavirus 2 (SARS-CoV-2) in Respiratory specimen | OMOP Extension |
| 586307 | Measurement of Severe acute respiratory syndrome coronavirus 2 (SARS-CoV-2) in Saliva | OMOP Extension |
| 705107 | Measurement of Severe acute respiratory syndrome coronavirus 2 (SARS-CoV-2) in Sample from nose | OMOP Extension |
| 586309 | Measurement of Severe acute respiratory syndrome coronavirus 2 (SARS-CoV-2) in Specified specimen | OMOP Extension |
| 756065 | Measurement of Severe acute respiratory syndrome coronavirus 2 (SARS-CoV-2) in Unspecified specimen | OMOP Extension |
| 704992 | Measurement of Severe acute respiratory syndrome coronavirus 2 (SARS-CoV-2) using Culture method | OMOP Extension |
| 705001 | Measurement of Severe acute respiratory syndrome coronavirus 2 (SARS-CoV-2) using Nucleic acid amplification technique | OMOP Extension |
| 705000 | Measurement of Severe acute respiratory syndrome coronavirus 2 (SARS-CoV-2) using Nucleic acid amplification technique in Blood | OMOP Extension |
| 756085 | Measurement of Severe acute respiratory syndrome coronavirus 2 (SARS-CoV-2) using Nucleic acid amplification technique in Respiratory specimen | OMOP Extension |
| 586308 | Measurement of Severe acute respiratory syndrome coronavirus 2 (SARS-CoV-2) using Nucleic acid amplification technique in Saliva | OMOP Extension |
| 705106 | Measurement of Severe acute respiratory syndrome coronavirus 2 (SARS-CoV-2) using Nucleic acid amplification technique in Sample from nose | OMOP Extension |
| 756084 | Measurement of Severe acute respiratory syndrome coronavirus 2 (SARS-CoV-2) using Nucleic acid amplification technique in Unspecified specimen | OMOP Extension |
| 704993 | Measurement of Severe acute respiratory syndrome coronavirus 2 (SARS-CoV-2) using Sequencing | OMOP Extension |
| 586516 | SARS-CoV-2 (COVID19) [Presence] in Unspecified specimen by Organism specific culture | LOINC |
| 723480 | SARS-CoV-2 (COVID19) Ab [Interpretation] in Serum or Plasma | LOINC |
| 586515 | SARS-CoV-2 (COVID19) Ab [Presence] in Serum or Plasma by Immunoassay | LOINC |
| 586522 | SARS-CoV-2 (COVID19) Ab [Units/volume] in Serum or Plasma by Immunoassay | LOINC |
| 706179 | SARS-CoV-2 (COVID19) Ab panel - Serum or Plasma by Immunoassay | LOINC |
| 723477 | SARS-CoV-2 (COVID19) Ag [Presence] in Respiratory specimen by Rapid immunoassay | LOINC |
| 706166 | SARS-CoV-2 (COVID19) E gene [Cycle Threshold #] in Unspecified specimen by NAA with probe detection | LOINC |
| 586523 | SARS-CoV-2 (COVID19) E gene [Presence] in Respiratory specimen by NAA with probe detection | LOINC |
| 586518 | SARS-CoV-2 (COVID19) E gene [Presence] in Serum or Plasma by NAA with probe detection | LOINC |
| 706174 | SARS-CoV-2 (COVID19) E gene [Presence] in Unspecified specimen by NAA with probe detection | LOINC |
| 723473 | SARS-CoV-2 (COVID19) IgA Ab [Presence] in Serum or Plasma by Immunoassay | LOINC |
| 586521 | SARS-CoV-2 (COVID19) IgA Ab [Presence] in Serum Plasma or Blood by Rapid immunoassay | LOINC |
| 723459 | SARS-CoV-2 (COVID19) IgA Ab [Units/volume] in Serum or Plasma by Immunoassay | LOINC |
| 757686 | SARS-CoV-2 (COVID19) IgA+IgM [Presence] in Serum or Plasma by Immunoassay | LOINC |
| 586527 | SARS-CoV-2 (COVID19) IgG Ab [Presence] in DBS by Immunoassay | LOINC |
| 723474 | SARS-CoV-2 (COVID19) IgG Ab [Presence] in Serum or Plasma by Immunoassay | LOINC |
| 706181 | SARS-CoV-2 (COVID19) IgG Ab [Presence] in Serum Plasma or Blood by Rapid immunoassay | LOINC |
| 706177 | SARS-CoV-2 (COVID19) IgG Ab [Units/volume] in Serum or Plasma by Immunoassay | LOINC |
| 706176 | SARS-CoV-2 (COVID19) IgG and IgM panel - Serum Plasma or Blood by Rapid immunoassay | LOINC |
| 723479 | SARS-CoV-2 (COVID19) IgG+IgM Ab [Presence] in Serum or Plasma by Immunoassay | LOINC |
| 723475 | SARS-CoV-2 (COVID19) IgM Ab [Presence] in Serum or Plasma by Immunoassay | LOINC |
| 706180 | SARS-CoV-2 (COVID19) IgM Ab [Presence] in Serum Plasma or Blood by Rapid immunoassay | LOINC |
| 706178 | SARS-CoV-2 (COVID19) IgM Ab [Units/volume] in Serum or Plasma by Immunoassay | LOINC |
| 706167 | SARS-CoV-2 (COVID19) N gene [Cycle Threshold #] in Unspecified specimen by NAA with probe detection | LOINC |
| 706157 | SARS-CoV-2 (COVID19) N gene [Cycle Threshold #] in Unspecified specimen by Nucleic acid amplification using CDC primer-probe set N1 | LOINC |
| 706155 | SARS-CoV-2 (COVID19) N gene [Cycle Threshold #] in Unspecified specimen by Nucleic acid amplification using CDC primer-probe set N2 | LOINC |
| 715272 | SARS-CoV-2 (COVID19) N gene [Presence] in Nasopharynx by NAA with probe detection | LOINC |
| 757678 | SARS-CoV-2 (COVID19) N gene [Presence] in Nose by NAA with probe detection | LOINC |
| 706161 | SARS-CoV-2 (COVID19) N gene [Presence] in Respiratory specimen by NAA with probe detection | LOINC |
| 586524 | SARS-CoV-2 (COVID19) N gene [Presence] in Respiratory specimen by Nucleic acid amplification using CDC primer-probe set N1 | LOINC |
| 586525 | SARS-CoV-2 (COVID19) N gene [Presence] in Respiratory specimen by Nucleic acid amplification using CDC primer-probe set N2 | LOINC |
| 586520 | SARS-CoV-2 (COVID19) N gene [Presence] in Serum or Plasma by NAA with probe detection | LOINC |
| 706175 | SARS-CoV-2 (COVID19) N gene [Presence] in Unspecified specimen by NAA with probe detection | LOINC |
| 706156 | SARS-CoV-2 (COVID19) N gene [Presence] in Unspecified specimen by Nucleic acid amplification using CDC primer-probe set N1 | LOINC |
| 706154 | SARS-CoV-2 (COVID19) N gene [Presence] in Unspecified specimen by Nucleic acid amplification using CDC primer-probe set N2 | LOINC |
| 757680 | SARS-CoV-2 (COVID19) neutralizing antibody [Presence] in Serum by pVNT | LOINC |
| 757679 | SARS-CoV-2 (COVID19) neutralizing antibody [Titer] in Serum by pVNT | LOINC |
| 723469 | SARS-CoV-2 (COVID19) ORF1ab region [Cycle Threshold #] in Respiratory specimen by NAA with probe detection | LOINC |
| 706168 | SARS-CoV-2 (COVID19) ORF1ab region [Cycle Threshold #] in Unspecified specimen by NAA with probe detection | LOINC |
| 723478 | SARS-CoV-2 (COVID19) ORF1ab region [Presence] in Respiratory specimen by NAA with probe detection | LOINC |
| 723464 | SARS-CoV-2 (COVID19) ORF1ab region [Presence] in Unspecified specimen by NAA with probe detection | LOINC |
| 723471 | SARS-CoV-2 (COVID19) RdRp gene [Cycle Threshold #] in Respiratory specimen by NAA with probe detection | LOINC |
| 723470 | SARS-CoV-2 (COVID19) RdRp gene [Cycle Threshold #] in Unspecified specimen by NAA with probe detection | LOINC |
| 706160 | SARS-CoV-2 (COVID19) RdRp gene [Presence] in Respiratory specimen by NAA with probe detection | LOINC |
| 706173 | SARS-CoV-2 (COVID19) RdRp gene [Presence] in Unspecified specimen by NAA with probe detection | LOINC |
| 586528 | SARS-CoV-2 (COVID19) RNA [Cycle Threshold #] in Respiratory specimen by NAA with probe detection | LOINC |
| 586529 | SARS-CoV-2 (COVID19) RNA [Cycle Threshold #] in Unspecified specimen by NAA with probe detection | LOINC |
| 715262 | SARS-CoV-2 (COVID19) RNA [Log #/volume] (viral load) in Unspecified specimen by NAA with probe detection | LOINC |
| 723476 | SARS-CoV-2 (COVID19) RNA [Presence] in Nasopharynx by NAA with non-probe detection | LOINC |
| 586526 | SARS-CoV-2 (COVID19) RNA [Presence] in Nasopharynx by NAA with probe detection | LOINC |
| 757677 | SARS-CoV-2 (COVID19) RNA [Presence] in Nose by NAA with probe detection | LOINC |
| 706163 | SARS-CoV-2 (COVID19) RNA [Presence] in Respiratory specimen by NAA with probe detection | LOINC |
| 715260 | SARS-CoV-2 (COVID19) RNA [Presence] in Saliva (oral fluid) by NAA with probe detection | LOINC |
| 715261 | SARS-CoV-2 (COVID19) RNA [Presence] in Saliva (oral fluid) by Sequencing | LOINC |
| 723463 | SARS-CoV-2 (COVID19) RNA [Presence] in Serum or Plasma by NAA with probe detection | LOINC |
| 706170 | SARS-CoV-2 (COVID19) RNA [Presence] in Unspecified specimen by NAA with probe detection | LOINC |
| 706158 | SARS-CoV-2 (COVID19) RNA panel - Respiratory specimen by NAA with probe detection | LOINC |
| 706169 | SARS-CoV-2 (COVID19) RNA panel - Unspecified specimen by NAA with probe detection | LOINC |
| 723467 | SARS-CoV-2 (COVID19) S gene [Cycle Threshold #] in Respiratory specimen by NAA with probe detection | LOINC |
| 723468 | SARS-CoV-2 (COVID19) S gene [Cycle Threshold #] in Unspecified specimen by NAA with probe detection | LOINC |
| 723465 | SARS-CoV-2 (COVID19) S gene [Presence] in Respiratory specimen by NAA with probe detection | LOINC |
| 586519 | SARS-CoV-2 (COVID19) S gene [Presence] in Serum or Plasma by NAA with probe detection | LOINC |
| 723466 | SARS-CoV-2 (COVID19) S gene [Presence] in Unspecified specimen by NAA with probe detection | LOINC |
| 586517 | SARS-CoV-2 (COVID19) whole genome [Nucleotide sequence] in Isolate by Sequencing | LOINC |
| 40218805 | Testing for SARS-CoV-2 in CDC laboratory | HCPCS |
| 40218804 | Testing for SARS-CoV-2 in non-CDC laboratory | HCPCS |

####

#### Appendix 3. Definitions and codes used for obesity and other comorbidities

| **Name** | **Definition** | **Included Codes** |
| --- | --- | --- |
| Asthma | People having any of the following:   - a drug exposure of Asthma therapy   - with age < 55 - Having all of the following criteria:   - at least 1 occurrences of a drug exposure of Asthma therapy   - where event starts between 365 days Before and 180 days Before index start date - a condition occurrence of Asthma - an observation of Asthma   with continuous observation of at least 0 days prior and 0 days after event index date and limit initial events to: earliest event per person. | <https://atlas.ohdsi.org/#/cohortdefinition/218> |
| Autoimmune condition | People having any of the following condition occurrence/observation:   - Type 1 Diabetes Mellitus Rheumatoid arthritis Psoriasis Psoriatic Arthritis Multiple sclerosis Systemic lupus erythematosus Addison's disease Graves' disease Sjogren's syndrome Hashimoto thyroiditis Myasthenia gravis Vasculitis Pernicious anemia Celiac disease Scleroderma Sarcoidosis Ulcerative colitis Crohn's disease   with continuous observation of at least 0 days prior and 0 days after event index date and limit initial events to: earliest event per person. | https://atlas.ohdsi.org/#/cohortdefinition/220 |
| Chronic kidney disease broad | People having any of the following:   - a condition occurrence of Chronic kidney disease   with continuous observation of at least 0 days prior and 0 days after event index date and limit initial events to: earliest event per person. | https://atlas.ohdsi.org/#/cohortdefinition/312 |
| Chronic obstructive pulmonary disease | People having any of the following:   - a drug exposure of COPD combo-therapy   - with age >= 55 - a drug exposure of COPD mono-therapy   - with age >= 55 - a condition occurrence of Chronic obstructive lung disease   with continuous observation of at least 0 days prior and 0 days after event index date and limit initial events to: earliest event per person. | https://atlas.ohdsi.org/#/cohortdefinition/219 |
| Dementia | People having any of the following:   - a condition occurrence of Dementia   with continuous observation of at least 0 days prior and 0 days after event index date and limit initial events to: earliest event per person. | https://atlas.ohdsi.org/#/cohortdefinition/226 |
| Heart disease | People having any of the following:   - a condition occurrence of Heart disease conditions   with continuous observation of at least 0 days prior and 0 days after event index date and limit initial events to: earliest event per person. | https://atlas.ohdsi.org/#/cohortdefinition/231 |
| Human immunodeficiency virus infection broad | People having any of the following:   - a condition occurrence of HIV excluding HIV-II   with continuous observation of at least 0 days prior and 0 days after event index date and limit initial events to: earliest event per person. | https://atlas.ohdsi.org/#/cohortdefinition/314 |
| Hypertension | People having any of the following:   - a condition occurrence of Hypertension   with continuous observation of at least 0 days prior and 0 days after event index date and limit initial events to: earliest event per person. | <https://atlas.ohdsi.org/#/cohortdefinition/227> |
| Malignant neoplasm excluding non-melanoma skin cancer | People having any of the following:   - a condition occurrence of Malignant neoplasms excluding non-melanoma skin cancer - an observation of Malignant neoplasms excluding non-melanoma skin cancer   with continuous observation of at least 0 days prior and 0 days after event index date and limit initial events to: earliest event per person. | https://atlas.ohdsi.org/#/cohortdefinition/222 |
| Obesity | People having any of the following:   - a measurement of BMI measurement with value as number between 30 and 60 (inclusive) - a condition occurrence of obesity diagnoses - an observation of obesity diagnoses - a measurement of body weight   - with value as number between 120 and 200 (inclusive)   - unit is any of: kilogram - an observation of body weight   - with value as number between 120 and 200 (inclusive)   - unit is any of: kilogram   with continuous observation of at least 0 days prior and 0 days after event index date and limit initial events to: **earliest event per person.**  Limit qualifying cohort to: **earliest event per person.** | https://atlas.ohdsi.org/#/cohortdefinition/224 |
| Pregnancy | People having any of the following:   - a condition occurrence of Pregnancy conditions procedures and observations^2^   - with age between 12 and 55 (inclusive)   - gender is any of: FEMALE - a procedure of Pregnancy conditions procedures and observations   - with age between 12 and 55 (inclusive)   - gender is any of: FEMALE - an observation of Pregnancy conditions procedures and observations   - with age between 12 and 55 (inclusive)   - gender is any of: FEMALE   with continuous observation of at least 0 days prior and 0 days after event index date and limit initial events to: **all events per person.**  Inclusion rules  Inclusion Criteria #1: At least 1 outcome 1 day after or end of observation before end of pregnancy post-2020  Having any of the following criteria:   - at least 1 occurrences of a condition occurrence of Birth Abortion Miscarriage^1^ - where event starts between 1 days After and 300 days After index start date - or at least 1 occurrences of an observation of Birth Abortion Miscarriage^1^ - where event starts between 1 days After and 300 days After index start date - or at least 1 occurrences of a procedure of Birth Abortion Miscarriage^1^ - where event starts between 1 days After and all days After index start date - or at least 1 occurrences of an observation period   - Having all of the following criteria:     - with the following event criteria:       - occurrence end is after 2020-01-01 - where event starts between all days Before and 0 days After index start date and event ends between 0 days After and 300 days After index start date   Inclusion Criteria #2: No outcome in the prior 60d  Having all of the following criteria:   - exactly 0 occurrences of a condition occurrence of Birth Abortion Miscarriage^1^ - where event starts between 60 days Before and 1 days Before index start date - and exactly 0 occurrences of a procedure of Birth Abortion Miscarriage^1^ - where event starts between 60 days Before and 1 days Before index start date - and exactly 0 occurrences of an observation of Birth Abortion Miscarriage^1^ - where event starts between 60 days Before and 1 days Before index start date   Limit qualifying cohort to: **all events per person.** | https://atlas.ohdsi.org/#/cohortdefinition/233 |
| Type 2 Diabetes Mellitus | People having any of the following:   - a condition occurrence of Type 2 Diabetes Mellitus - an observation of History of diabetes   with continuous observation of at least 0 days prior and 0 days after event index date and limit initial events to: earliest event per person. | https://atlas.ohdsi.org/#/cohortdefinition/311 |

#### Appendix 4. Table 3: Selected comorbidities in % (95%CI) among patients living with and without obesity diagnosed with COVID-19, by database

|  | **SIDIAP**  **(Spain)** | | | **CPRD**  **(UK)** | | | **CUIMC**  **(US)** | | | **IQVIA-Open**  **Claims (US)** | | | **STARR-OMOP**  **(US)** | | | **VA-OMOP**  **(US)** | | |
| --- | --- | --- | --- | --- | --- | --- | --- | --- | --- | --- | --- | --- | --- | --- | --- | --- | --- | --- |
|  | With obesity | Without obesity | SMD | With obesity | Without obesity | SMD | With obesity | Without obesity | SMD | With obesity | Without obesity | SMD | With obesity | Without obesity | SMD | With obesity | Without obesity | SMD |
| n | 36 409 | 854 649 | NA | 976 | 1360 | NA | 3446 | 5073 | NA | 154 325 | 311 866 | NA | 1157 | 2171 | NA | 11 546 | 13 066 | NA |
| **Respiratory** |  |  |  |  |  |  |  |  |  |  |  |  |  |  |  |  |  |  |
| Apnea | 7.5  (7.2-7.8) | 1.5  (1.5-1.5) | 0.21 | - | - | - | 7.1 (6.2-8.0) | 1.1 (0.8-1.4) | 0.22 | 12.4 (12.2-12.6) | 2.6 (2.5-2.7) | 0.27 | 20.3 (18.0-22.6) | 6.7 (5.6-7.8) | 0.29 | 37.5 (36.6-38.4) | 11.7 (11.1-12.3) | 0.44 |
| Asthma | 7.4 (7.1-7.7) | 5.6  (5.6-5.6) | 0.05 | 20.6 (18.1-23.1) | 10.4 (8.8-12.0) | 0.20 | 22.1 (20.7-23.5) | 10.9 (10.0-11.8) | 0.22 | 25.5 (25.3-25.7) | 14.5 (14.4-14.6) | 0.20 | 19.5 (17.2-21.8) | 10.8 (9.5-12.1) | 0.17 | 16.0 (15.3-16.7) | 8.3 (7.8-8.8) | 0.17 |
| COPD | 22.2 (21.8-22.6) | 8.7  (8.6-8.8) | 0.27 | 10.1 (8.2-12.0) | 6.5 (5.2-7.8) | 0.09 | 12.8 (11.7-13.9) | 6.0 (5.3-6.7) | 0.17 | 15.7 (15.5-15.9) | 13.7 (13.6-13.8) | 0.04 | 8.6 (7.0-10.2) | 4.8 (3.9-5.7) | 0.11 | 30.8 (30.0-31.6) | 22.6 (21.9-23.3) | 0.13 |
| Idiopathic pulmonary fibrosis | 0.1 (0.1-0.1) | 0.0 (0.0-0.0) | 0.03 | - | - | - | 0.3 (0.1-0.5) | - | - | 0.1 (0.1-0.1) | 0.1 (0.1-0.1) | 0.00 | - | - | - | 0.2 (0.1-0.3) | 0.1 (0.0-0.2) | 0.02 |
| **Metabolic** |  |  |  |  |  |  |  |  |  |  |  |  |  |  |  |  |  |  |
| Type 2 Diabetes | 17.7 (17.3-18.1) | 4.0 (4.0-4.0) | 0.32 | 27.7 (24.9-30.5) | 7.0 (5.6-8.4) | 0.40 | 34.8 (33.2-36.4) | 13.3 (12.4-14.2) | 0.37 | 49.8 (49.6-50.0) | 29.3 (29.1-29.5) | 0.30 | 26.6 (24.1-29.1) | 8.9 (7.7-10.1) | 0.34 | 53.6 (52.7-54.5) | 23.0 (22.3-23.7) | 0.47 |
| Hyperlipidemia | 22.2 (21.8-22.6) | 11.6 (11.5-11.7) | 0.20 |  | -- | - | 26.6 (25.1-28.1) | 11.2 (10.3-12.1) | 0.28 | 36.6 (36.4-36.8) | 22.6 (22.5-22.7) | 0.22 | 36.2 (33.4-39.0) | 19.7 (18.0-21.4) | 0.26 | 56.8 (55.9-57.7) | 34.9 (34.1-35.7) | 0.32 |
| **Cardiovascular** |  |  |  |  |  |  |  |  |  |  |  |  |  |  |  |  |  |  |
| Atrial fibrillation | 7.8  (7.5-8.1) | 2.6  (2.6-2.6) | 0.17 | - | - | - | 6.8  (6.0-7.6) | 2.5  (2.1-2.9) | 0.14 | 7.4  (7.3-7.5) | 5.9  (5.8-6.0) | 0.04 | 7.5  (6.0-9.0) | 4.5  (3.6-5.4) | 0.09 | 10.9 (10.3-11.5) | 7.3  (6.9-7.7) | 0.09 |
| Cerebrovascular disease | 2.0  (1.9-2.1) | 1.0  (1.0-1.0) | 0.06 | 1.0  (0.6-2.0) | 1.4  (0.8-2.0) | -0.01 | 4.5  (3.8-5.2) | 2.3  (1.9-2.7) | 0.09 | 5.3  (5.2-5.4) | 4.3  (4.2-4.4) | 0.03 | 3.1  (2.1-4.1) | 2.4  (1.8-3.0) | 0.03 | 5.4  (5.0-5.8) | 3.8  (3.5-4.1) | 0.05 |
| Heart disease | 24.1 (23.7-24.5) | 10.3 (10.2-10.4) | 0.26 | 25.7 (23.0-28.4) | 16.2 (14.2-18.2) | 0.17 | 49.1 (47.4-50.8) | 25.8 (24.6-27.0) | 0.35 | 54.9 (54.7-55.1) | 41.5 (41.3-41.7) | 0.19 | 36.0 (33.2-38.8) | 22.2 (20.5-23.9) | 0.22 | 59.5 (58.6-60.4) | 39.8 (39.0-40.6) | 0.28 |
| Heart failure | 6.0  (5.8-6.2) | 1.5  (1.5-1.5) | 0.17 | - | - | - | 8.4  (7.5-9.3) | 2.9  (2.4-3.4) | 0.17 | 12.1 (11.9-12.3) | 7.5  (7.4-7.6) | 0.11 | 7.1  (5.6-8.6) | 3.1  (2.4-3.8) | 0.13 | 12.7 (12.1-13.3) | 6.7  (6.3-7.1) | 0.14 |
| Hypertension | 29.7 (29.2-30.2) | 11.8 (11.7-11.9) | 0.32 | 31.7 (28.8-34.6) | 15.7 (13.8-17.6) | 0.27 | 61.5 (59.9-63.1) | 29.8 (28.5-31.1) | 0.47 | 73.1 (72.9-73.3) | 52.2 (52.0-52.4) | 0.31 | 54.6 (51.7-57.5) | 25.7 (23.9-27.5) | 0.44 | 80.6 (79.9-81.3) | 53.3 (52.4-54.2) | 0.43 |
| Ischemic heart disease | 5.8  (5.6-6.0) | 2.2  (2.2-2.2) | 0.13 | - | - | 0.01 | 5.5 (4.7-6.3) | 2.1  (1.7-2.5) | 0.13 | 6.1  (6.0-6.2) | 4.1  (4.0-4.2) | 0.06 | 4.8  (3.6-6.0) | 2.8  (2.1-3.5) | 0.07 | 10.1 (9.6-10.6) | 6.1  (5.7-6.5) | 0.10 |
| Peripheral vascular disease | 2.8 (2.6-3.0) | 1.2 (1.2-1.2) | 0.08 | - | - |  | 4.1 (3.4-4.8) | 1.8 (1.4-2.2) | 0.10 | 8.5 (8.4-8.6) | 5.5 (5.4-5.6) | 0.08 | 3.2 (2.2-4.2) | 1.6 (1.1-2.1) | 0.07 | 8.1 (7.6-8.6) | 5.2 (4.8-5.6) | 0.08 |
| **Mental health and neurologic** |  |  |  |  |  |  |  |  |  |  |  |  |  |  |  |  |  |  |
| Anxiety | 31.6 (31.1-32.1) | 26.2 (26.1-26.3) | 0.08 | - | - | - | 9.8 (8.8-10.8) | 5.0 (4.4-5.6) | 0.13 | 13.3 (13.1-13.5) | 9.5 (9.4-9.6) | 0.08 | 16.3 (14.2-18.4) | 9.4 (8.2-10.6) | 0.15 | 37.2 (36.3-38.1) | 28.0 (27.2-28.8) | 0.14 |
| Dementia | 7.1 (6.8-7.4) | 4.0 (4.0-4.0) | 0.10 | 6.6 (5.0-8.2) | 8.5 (7.0-10.0) | -0.05 | 6.9 (6.1-7.7) | 4.5 (3.9-5.1) | 0.07 | 8.3 (8.2-8.4) | 14.0 (13.9-14.1) | -0.13 | 1.3 (0.6-2.0) | 0.7 (0.3-1.1) | 0.04 | 8.7 (8.2-9.2) | 9.3 (8.8-9.8) | -0.01 |
| Depression | 6.5 (6.2-6.8) | 4.7 (4.7-4.7) | 0.06 | 5.3 (3.9-6.7) | 4.0 (3.0-5.0) | 0.04 | 14.8 (13.6-16.0) | 5.2 (4.6-5.8) | 0.23 | 12.5 (12.3-12.7) | 8.0 (7.9-8.1) | 0.11 | 15.1 (13.0-17.2) | 7.1 (6.0-8.2) | 0.18 | 26.8 (26.0-27.6) | 20.3 (19.6-21.0) | 0.11 |
| **Others** |  |  |  |  |  |  |  |  |  |  |  |  |  |  |  |  |  |  |
| Anemia | 15.9 (15.5-16.3) | 10.0 (9.9-10.1) | 0.12 | - | - | - | 13.5 (12.4-14.6) | 6.3 (5.6-7.0) | 0.17 | 18.2 (18.0-18.4) | 13.8 (13.7-13.9) | 0.08 | 16.9 (14.7-19.1) | 11.1 (9.8-12.4) | 0.12 | 17.8 (17.1-18.5) | 13.7 (13.1-14.3) | 0.08 |
| Autoimmune condition | 9.4 (9.1-9.7) | 5.6 (5.6-5.6) | 0.10 | 14.7 (12.5-16.9) | 9.9 (8.3-11.5) | 0.10 | 23.7 (22.3-25.1) | 10.5 (9.7-11.3) | 0.25 | 30.2 (30.0-30.4) | 18.5 (18.4-18.6) | 0.19 | 15.8 (13.7-17.9) | 10.1 (8.8-11.4) | 0.12 | 27.8 (27.0-28.6) | 14.7 (14.1-15.3) | 0.23 |
| Chronic kidney disease | 12.0 (11.7-12.3) | 4.4 (4.4-4.4) | 0.20 | 17.3 (14.9-19.7) | 10.6 (9.0-12.2) | 0.14 | 20.3 (19.0-21.6) | 8.3 (7.5-9.1) | 0.25 | 23.1 (22.9-23.3) | 16.0 (15.9-16.1) | 0.13 | 18.3 (16.1-20.5) | 7.4 (6.3-8.5) | 0.23 | 26.8 (26.0-27.6) | 16.2 (15.6-16.8) | 0.18 |
| Chronic liver disease | 1.4 (1.3-1.5) | 1.0 (1.0-1.0) | 0.03 | - | - | - | 1.8 (1.4-2.2) | 0.9 (0.6-1.2) | 0.05 | 1.4 (1.3-1.5) | 1.0 (1.0-1.0) | 0.03 | 4.8 (3.6-6.0) | 2.0 (1.4-2.6) | 0.11 | 4.9 (4.5-5.3) | 3.9 (3.6-4.2) | 0.03 |
| History of cancer | 10.1  (9.8-10.4) | 6.0 (5.9-6.1) | 0.11 | 9.9 (8.0-11.8) | 8.2 (6.7-9.7) | 0.04 | 22.1 (20.7-23.5) | 13.0 (12.1-13.9) | 0.17 | 17.0 (16.8-17.2) | 14.9 (14.8-15.0) | 0.04 | 28.3 (25.7-30.9) | 22.2 (20.5-23.9) | 0.10 | 24.8 (24.0-25.6) | 20.1 (19.4-20.8) | 0.08 |
| Pregnancy | 0.7 (0.6-0.8) | 0.6 (0.6-0.6) | 0.01 | 0.9 (0.3-1.5) | 0.6 (0.2-1.0) | 0.02 | 6.7 (5.9-7.5) | 3.1 (2.6-3.6) | 0.12 | 2.5 (2.4-2.6) | 1.9 (1.9-1.9) | 0.03 | 1.6 (0.9-2.3) | 1.6 (1.1-2.1) | 0.00 | 0.2 (0.1-0.3) | 0.2 (0.1-0.3) | 0.00 |
| Human immunodeficiency virus infection | 0.0  (0.0-0.0) | 0.1 (0.1-0.1) | -0.04 | - | - |  | 1.8 (1.4-2.2) | 1.1 (0.8-1.4) | 0.04 | 1.0 (1.0-1.0) | 1.0 (1.0-1.0) | 0.00 | - | 0.6 (0.3-0.9) | - | 1.3 (1.1-1.5) | 1.9 (1.7-2.1) | -0.03 |

**Notes:** - data not available or below the minimum cell count required (5 individuals). SMD<0 means the prevalence was greater in COVID-19 patients living without obesity, SMD>0 means the prevalence was greater in COVID-19 patients living with obesity. A |SMD| >0.1 indicates a meaningful difference in the prevalence of a given condition.

**Abbreviations:** CI: Confidence Interval; COVID-19: Coronavirus Disease 2019; CPRD: Clinical Practice Research Datalink; CUIMC: Columbia University Irving Medical Center; NA: Not Applicable; SIDIAP: Information System for Research in Primary Care; SMD: Standardized Mean Difference; STARR-OMOP: Stanford Medicine Research Data Repository; VA-OMOP: United States Department of Veterans Affairs.

####

####

#### Appendix 5. Table 4: Selected comorbidities, in % (95%CI) among patients living with and without obesity hospitalized with COVID-19, by database

|  | **SIDIAP**  **(Spain)** | | | **CUIMC**  **(US)** | | | **IQVIA-OpenClaims (US)** | | | **STARR-OMOP**  **(US)** | | | **VA-OMOP**  **(US)** | | |
| --- | --- | --- | --- | --- | --- | --- | --- | --- | --- | --- | --- | --- | --- | --- | --- |
|  | With  obesity | Without obesity | SMD | With  obesity | Without obesity | SMD | With  obesity | Without obesity | SMD | With  obesity | Without obesity | SMD | With  obesity | Without obesity | SMD |
| n | 8403 | 9794 | NA | 1408 | 1192 | NA | 50 863 | 82 228 | NA | 274 | 341 | NA | 2918 | 2592 | NA |
| **Respiratory** |  |  |  |  |  |  |  |  |  |  |  |  |  |  |  |
| Apnea | 10.8 (10.1-11.5) | 3.0 (2.7-3.3) | 0.22 | 6.2 (4.9-7.5) | 1.4 (0.7-2.1) | 0.18 | 15.7 (15.4-16.0) | 3.4 (3.3-3.5) | 0.30 | 28.5 (23.2-33.8) | 11.4 (8.0-14.8) | 0.31 | 38.7 (36.9-40.5) | 11.5 (10.3-12.7) | 0.47 |
| Asthma | 6.3 (5.8-6.8) | 4.3 (3.9-4.7) | 0.06 | 22.3 (20.1-24.5) | 13.3 (11.4-15.2) | 0.17 | 24.3 (23.9-24.7) | 12.7 (12.5-12.9) | 0.21 | 21.5 (16.6-26.4) | 13.5 (9.9-17.1) | 0.15 | 14.2 (12.9-15.5) | 7.3 (6.3-8.3) | 0.16 |
| COPD | 33.2 (32.2-34.2) | 20.8 (20.0-21.6) | 0.20 | 18.7 (16.7-20.7) | 13.9 (11.9-15.9) | 0.09 | 23.4 (23.0-23.8) | 22.5 (22.2-22.8) | 0.02 | 12.8 (8.8-16.8) | 9.1 (6.0-12.2) | 0.08 | 43.9 (42.1-45.7) | 41.4 (39.5-43.3) | 0.04 |
| Idiopathic pulmonary fibrosis | 0.1 (0.0-0.2) | 0.1 (0.0-0.2) | 0.00 | 0.5 (0.1-0.9) | - |  | 0.2 (0.2-0.2) | 0.1 (0.1-0.1) | 0.02 | - | - |  | 0.2 (0.0-0.4) | - |  |
| **Metabolic** |  |  |  |  |  |  |  |  |  |  |  |  |  |  |  |
| Type 2 Diabetes | 26.1 (25.2-27.0) | 10.7 (10.1-11.3) | 0.29 | 47.7 (45.1-50.3) | 30.9 (28.3-33.5) | 0.25 | 64.0 (63.6-64.4) | 44.9 (44.6-45.2) | 0.28 | 29.2 (23.8-34.6) | 12.0 (8.6-15.4) | 0.31 | 67.8 (66.1-69.5) | 42.1 (40.2-44.0) | 0.38 |
| Hyperlipidemia | 28.6 (27.6-29.6) | 21.0 (20.2-21.8) | 0.12 | 29.8 (27.4-32.2) | 19.0 (16.8-21.2) | 0.18 | 42.8 (42.4-43.2) | 29.3 (29.0-29.6) | 0.20 | 43.8 (37.9-49.7) | 32.6 (27.6-37.6) | 0.16 | 62.6 (60.8-64.4) | 48.8 (46.9-50.7) | 0.20 |
| **Cardiovascular** |  |  |  |  |  |  |  |  |  |  |  |  |  |  |  |
| Atrial fibrillation | 11.2 (10.5-11.9) | 6.4 (5.9-6.9) | 0.12 | 9.0 (7.5-10.5) | 5.5 (4.2-6.8) | 0.10 | 12.2 (11.9-12.5) | 10.4 (10.2-10.6) | 0.04 | 11.3 (7.6-15.0) | 6.7 (4.0-9.4) | 0.11 | 17.3 (15.9-18.7) | 14.3 (13.0-15.6) | 0.06 |
| Cerebrovascular disease | 2.6 (2.3-2.9) | 2.3 (2.0-2.6) | 0.01 | 5.6 (4.4-6.8) | 3.7 (2.6-4.8) | 0.06 | 8.2 (8.0-8.4) | 7.4 (7.2-7.6) | 0.02 | 5.5 (2.8-8.2) | 6.5 (3.9-9.1) | -0.03 | 8.4 (7.4-9.4) | 7.3 (6.3-8.3) | 0.03 |
| Heart disease | 34.7 (33.7-35.7) | 22.3 (21.5-23.1) | 0.20 | 59.0 (56.4-61.6) | 43.2 (40.4-46.0) | 0.23 | 69.8 (69.4-70.2) | 59.8 (59.5-60.1) | 0.15 | 44.5 (38.6-50.4) | 35.8 (30.7-40.9) | 0.13 | 74.4 (72.8-76.0) | 65.4 (63.6-67.2) | 0.14 |
| Heart failure | 9.2 (8.6-9.8) | 4.0 (3.6-4.4) | 0.15 | 13.5 (11.7-15.3) | 6.9 (5.5-8.3) | 0.16 | 21.5 (21.1-21.9) | 14.7 (14.5-14.9) | 0.13 | 12.0 (8.2-15.8) | 6.5 (3.9-9.1) | 0.13 | 22.8 (21.3-24.3) | 16.9 (15.5-18.3) | 0.10 |
| Hypertension | 36.9 (35.9-37.9) | 23.9 (23.1-24.7) | 0.20 | 72.0 (69.7-74.3) | 56.0 (53.2-58.8) | 0.24 | 86.2 (85.9-86.5) | 73.0 (72.7-73.3) | 0.23 | 60.9 (55.1-66.7) | 43.1 (37.8-48.4) | 0.26 | 91.0 (90.0-92.0) | 81.1 (79.6-82.6) | 0.20 |
| Ischemic heart disease | 9.1 (8.5-9.7) | 6.3 (5.8-6.8) | 0.07 | 8.8 (7.3-10.3) | 5.5 (4.2-6.8) | 0.09 | 10.9 (10.6-11.2) | 8.4 (8.2-8.6) | 0.06 | 7.7 (4.5-10.9) | 5.3 (2.9-7.7) | 0.07 | 16.4 (15.1-17.7) | 13.2 (11.9-14.5) | 0.06 |
| Peripheral vascular disease | 4.8 (4.3-5.3) | 3.5 (3.1-3.9) | 0.05 | 5.8 (4.6-7.0) | 4.0 (2.9-5.1) | 0.06 | 13.3 (13.0-13.6) | 9.7 (9.5-9.9) | 0.08 | 3.6 (1.4-5.8) | - |  | 14.4 (13.1-15.7) | 11.8 (10.6-13.0) | 0.05 |
| **Mental health and neurologic** |  |  |  |  |  |  |  |  |  |  |  |  |  |  |  |
| Anxiety | 26.7 (25.8-27.6) | 22.0 (21.2-22.8) | 0.08 | 8.7 (7.2-10.2) | 3.7 (2.6-4.8) | 0.15 | 12.9 (12.6-13.2) | 9.5 (9.3-9.7) | 0.08 | 21.2 (16.4-26.0) | 17.6 (13.6-21.6) | 0.06 | 34.3 (32.6-36.0) | 27.4 (25.7-29.1) | 0.11 |
| Dementia | 6.3 (5.8-6.8) | 5.8 (5.3-6.3) | 0.01 | 12.9 (11.1-14.7) | 14.8 (12.8-16.8) | -0.04 | 11.7 (11.4-12.0) | 19.9 (19.6-20.2) | -0.16 | - | - |  | 16.3 (15.0-17.6) | 24.2 (22.6-25.8) | -0.14 |
| Depression | 5.9 (5.4-6.4) | 5.0 (4.6-5.4) | 0.03 | 17.0 (15.0-19.0) | 7.1 (5.6-8.6) | 0.22 | 12.7 (12.4-13.0) | 8.4 (8.2-8.6) | 0.10 | 18.2 (13.6-22.8) | 11.4 (8.0-14.8) | 0.14 | 23.2 (21.7-24.7) | 19.4 (17.9-20.9) | 0.07 |
| **Others** |  |  |  |  |  |  |  |  |  |  |  |  |  |  |  |
| Anemia | 17.6 (16.8-18.4) | 13.4 (12.7-14.1) | 0.08 | 16.8 (14.8-18.8) | 12.1 (10.2-14.0) | 0.09 | 25.1 (24.7-25.5) | 22.0 (21.7-22.3) | 0.05 | 25.5 (20.3-30.7) | 22.3 (17.9-26.7) | 0.05 | 28.2 (26.6-29.8) | 29.1 (27.4-30.8) | -0.01 |
| Autoimmune condition | 11.0 (10.3-11.7) | 7.8 (7.3-8.3) | 0.08 | 27.2 (24.9-29.5) | 13.8 (11.8-15.8) | 0.24 | 37.7 (37.3-38.1) | 24.9 (24.6-25.2) | 0.20 | 17.2 (12.7-21.7) | 9.4 (6.3-12.5) | 0.16 | 36.8 (35.1-38.5) | 24.7 (23.0-26.4) | 0.19 |
| Chronic kidney disease | 18.0 (17.2-18.8) | 11.6 (11.0-12.2) | 0.13 | 30.0 (27.6-32.4) | 18.3 (16.1-20.5) | 0.20 | 37.2 (36.8-37.6) | 29.4 (29.1-29.7) | 0.12 | 27.4 (22.1-32.7) | 13.8 (10.1-17.5) | 0.24 | 42.1 (40.3-43.9) | 33.7 (31.9-35.5) | 0.12 |
| Chronic liver disease | 1.7 (1.4-2.0) | 2.0 (1.7-2.3) | -0.02 | 2.9 (2.0-3.8) | 2.4 (1.5-3.3) | 0.02 | 2.2 (2.1-2.3) | 2.0 (1.9-2.1) | 0.01 | 5.8 (3.0-8.6) | 2.9 (1.1-4.7) | 0.10 | 7.8 (6.8-8.8) | 8.0 (7.0-9.0) | -0.01 |
| History of cancer | 14.6 (13.8-15.4) | 13.7 (13.0-14.4) | 0.02 | 23.2 (21.0-25.4) | 19.0 (16.8-21.2) | 0.07 | 22.4 (22.0-22.8) | 21.9 (21.6-22.2) | 0.01 | 33.6 (28.0-39.2) | 41.1 (35.9-46.3) | -0.11 | 32.4 (30.7-34.1) | 33.3 (31.5-35.1) | -0.01 |
| Pregnancy | 0.6 (0.4-0.8) | 0.7 (0.5-0.9) | -0.01 | 7.2 (5.8-8.6) | 2.9 (1.9-3.9) | 0.14 | 1.9 (1.8-2.0) | 1.6 (1.5-1.7) | 0.02 | - | - |  | - | - |  |
| Human immunodeficiency virus infection | - | 0.1 (0.0-0.2) |  | 1.6 (0.9-2.3) | 2.2 (1.4-3.0) | -0.03 | 1.3 (1.2-1.4) | 1.4 (1.3-1.5) | -0.01 | - | - |  | 1.5 (1.1-1.9) | 2.6 (2.0-3.2) | -0.05 |

**Notes:** - data not available or below the minimum cell count required (5 individuals). SMD<0 means the prevalence was greater in COVID-19 patients living without obesity, SMD>0 means the prevalence was greater in COVID-19 patients living with obesity. A |SMD| >0.1 indicates a meaningful difference in the prevalence of a given condition.

**Abbreviations:** CI: Confidence Interval; COVID-19: Coronavirus Disease 2019; CPRD: Clinical Practice Research Datalink; CUIMC: Columbia University Irving Medical Center; NA: Not Applicable; SIDIAP: Information System for Research in Primary Care; SMD: Standardized Mean Difference; STARR-OMOP: Stanford Medicine Research Data Repository; VA-OMOP: United States Department of Veterans Affairs.
